# Immunomodulatory Role of the Extracellular Matrix Within the Liver Disease Microenvironment

**DOI:** 10.3389/fimmu.2020.574276

**Published:** 2020-11-11

**Authors:** Claire E. McQuitty, Roger Williams, Shilpa Chokshi, Luca Urbani

**Affiliations:** ^1^Institute of Hepatology, Foundation for Liver Research, London, United Kingdom; ^2^Faculty of Life Sciences & Medicine, King’s College London, London, United Kingdom

**Keywords:** extracellular matrix, extracellular matrix–damage-associated molecular patterns, fibrosis, chronic liver disease, microenvironment, inflammation, immunomodulation

## Abstract

Chronic liver disease when accompanied by underlying fibrosis, is characterized by an accumulation of extracellular matrix (ECM) proteins and chronic inflammation. Although traditionally considered as a passive and largely architectural structure, the ECM is now being recognized as a source of potent damage-associated molecular pattern (DAMP)s with immune-active peptides and domains. In parallel, the ECM anchors a range of cytokines, chemokines and growth factors, all of which are capable of modulating immune responses. A growing body of evidence shows that ECM proteins themselves are capable of modulating immunity either directly *via* ligation with immune cell receptors including integrins and TLRs, or indirectly through release of immunoactive molecules such as cytokines which are stored within the ECM structure. Notably, ECM deposition and remodeling during injury and fibrosis can result in release or formation of ECM-DAMPs within the tissue, which can promote local inflammatory immune response and chemotactic immune cell recruitment and inflammation. It is well described that the ECM and immune response are interlinked and mutually participate in driving fibrosis, although their precise interactions in the context of chronic liver disease are poorly understood. This review aims to describe the known pro-/anti-inflammatory and fibrogenic properties of ECM proteins and DAMPs, with particular reference to the immunomodulatory properties of the ECM in the context of chronic liver disease. Finally, we discuss the importance of developing novel biotechnological platforms based on decellularized ECM-scaffolds, which provide opportunities to directly explore liver ECM-immune cell interactions in greater detail.

## Introduction

The extracellular matrix (ECM) constitutes a complex network of proteins which make up the overall architecture of an organ. It acts as a structural support, imparts mechano-elastic properties and delivers environmental cues to influence cellular proliferation, survival, shape, migration and differentiation and plays an important role in modulating tissue homeostasis, remodeling and regeneration (1–3). The ECM network can be broadly divided into different families of proteins based on their structure: collagens, elastin, cell-adhesion glycoproteins, glycosaminoglycans (GAGs), proteoglycans, and matricellular proteins. GAGs are long, unbranched chains of repeating disaccharide units; proteoglycans are formed by GAGs which are bound to core proteins. Matricellular proteins are non-structural ECM-associated proteins with regulatory activity that are released and/or incorporated into the ECM particularly during development and in response to injury (4). The ECM also binds a large number of ECM-associated molecules including growth factors, cytokines, and chemokines (5), which can bind to numerous ECM proteins (5), discussed in detail in the section “*ECM protein association with growth factors, cytokines and chemokines*”.

The ECM is ubiquitous in all tissues throughout the body; however, the proportions, specific composition and organization of ECM proteins differs organ-to-organ, resulting in a unique landscape which provides a set of properties integral to the organs’ function. Berger et al., highlighted this in a recent study using proteomics to compare the ECM of decellularized porcine pancreas, small intestine, and lung. The study showed clear differences in the ECM signature between decellularized organs and show that organ specific ECM landscapes drive spontaneously differentiating human induced pluripotent stem cells (hiPSCs) toward different lineages in an organ- dependent manner, elegantly revealing that the ECM possesses organ-specific biological cues (6).

In this review, we highlight the importance of the intricate ECM-immune cell interactions in the liver and during liver fibrosis and describe the known immunomodulatory activities of individual ECM molecules and fragments in chronic liver disease (CLD)s.

## ECM in the Healthy Liver

In the healthy liver, the ECM comprises up to 10% of the total volume (7) and is largely restricted to the portal tracts, sinusoidal walls and the central vein (8).

An in-depth proteomics analysis of decellularized human liver ECM by Naba et al., identified more than 150 distinct ECM proteins which make up the healthy human liver matrisome (9). Among these are: (1) a large and diverse number of collagens, including transmembrane-, network-forming-, fibrillar and fibril-associated collagens; (2) 44 ECM glycoproteins, the most abundant of which include fibrinogens and fibronectins; (3) 11 proteoglycans, including the small leucine-rich repeat proteoglycans: biglycan, decorin, and lumican; several understudied proteins: proteoglycan-2, -3, and -4, and versican; (4) a large number of proteins associated with the formation of elastic fibres; and (5) the matricellular proteins – periostin, tenascin-C and -X. Further details regarding these ECM proteins found in the healthy human liver matrisome can be found in a recent review by Arteel et al. (10).

In homeostasis, the composition of the ECM is constantly fine-tuned by a series of synthesis and degradation events. Hepatocytes, liver sinusoidal endothelial cells (LSECs), hepatic stellate cells (HSCs) and Kupffer cells (KCs) are all capable of synthesising ECM proteins (11), as well as matrix metalloproteinase (MMP)s and their inhibitors, tissue-inhibitors of MMPs (TIMPs) which can degrade most ECM proteins (12) and contribute to ECM remodeling and turnover in homeostasis and disease. MMPs are considered major contributors to ECM remodeling. They are secreted as latent zymogens, requiring activation, and are marginally expressed in the healthy liver, but expressed immediately after hepatic injury and thought to be involved in fibrosis resolution (12). Indeed, several rodent models of CLD show enhanced MMP expression linked to amelioration of fibrosis. For example, Kim et al. show that in a mouse model of CCl_4_ induced liver fibrosis, enhanced MMP-13 expression promoted recovery from liver fibrosis (13). Similarly, Siller-Lopez and colleagues showed that treatment with human MMP-8 showed amelioration of liver fibrosis in a CCl_4_ treated bile-duct ligation (BDL) rat model of liver fibrosis (14).

Careful management of ECM remodeling is required for tissue regeneration and wound healing and is crucial for maintaining homeostasis. Indeed, if the balance of synthesis and degradation is perturbed, e.g., following injury or insult, the liver can progress towards increased scar formation/fibrosis, during which ECM synthesis greatly outweighs degradation resulting in excessive accumulation of ECM proteins. HSCs are central participants in this fibrogenic processes. Local inflammatory signals can activate quiescent HSCs to a highly proliferative phenotype driving their transdifferentiation into myofibroblasts, which characteristically increase ECM secretion, this process underlies the development and progression of liver fibrosis (15). The mechanisms of HSC activation are complex and multi-factorial and can be characterized as: “initiation”, in which increased susceptibility to a range of extracellular signals promotes expression of growth factor receptors, modulation of growth factor signaling and transdifferentiation to a contractile and fibrogenic phenotype; and “perpetuation”, in which a series of positive feed-back loops from the ECM amplify this activated phenotype. Indeed, HSCs can interact with ECM proteins *via* α_v_β_3_ integrin which binds to (arginine- glycine- aspartic acid) RGD domains on ECM proteins. α_v_β_3_ integrin expression is upregulated during liver fibrosis development and progression (16) and ECM-α_v_β_3_ interactions have been suggested to support HSC proliferation (17). Initiation and perpetuation of HSC activation is modulated by complex cross-talk between liver cells, infiltrating immune cells and the ECM (18, 19). Finally, the ECM directly influences cell behaviour, including immune cells which can interact with ECM proteins through cell surface receptors such as integrins which activate intracellular signaling pathways (2), cell migration (1) and proliferation (4).

In summary, the interplay between the ECM, liver cells and immune cells helps maintain a balance between injury and repair. Perturbation of this equilibrium, for example by toxic injuries such as alcohol, can drive liver fibrogenesis which is characterized by progressive accumulation of ECM proteins coupled with chronic inflammation.

## ECM in Chronic Liver Disease

The progression of CLDs, including alcoholic liver disease (ALD), non-alcoholic fatty liver disease (NAFLD), non-alcoholic steatohepatitis (NASH), and Hepatitis B and C (HBV/HCV), is associated with the development of fibrosis. In these diseases, active remodeling of the ECM proteins, drives dramatic changes in the ECM landscape and the release of ECM-associated bioactive molecules (e.g., growth factors and cytokines). In addition, fragmented ECM proteins, generated upon remodeling or during injury, are recognized by immune cells as damage-associated molecular patterns (DAMPs), termed ECM-DAMPs.

Generally, liver fibrosis involves increased ECM protein synthesis and deposition, particularly of fibrillar collagens type I, which can be 8-fold higher than in healthy liver (20), collagen type III, fibronectin, and laminin (21) which are deposited in the Space of Disse, forming a dense matrix. If unresolved, a proportion of patients with fibrosis can progress to cirrhosis and/or hepatocellular carcinoma (HCC), the rate of which is associated with the underlying aetiology (22, 23). The reasons for these differences are not well understood but interestingly the stage of fibrosis and the causative aetiology seem to differ in the composition and organization of ECM components. This is detailed in **Table 1**. For example, fibrotic stage-specific changes in ECM composition during chronic HCV infection correlates with progressive accumulation of collagens I and II, followed by elastin over expression (28). Similarly, Daneshgar et al. reported upregulation of MMPs 23B and 28 and versican, in decellularized human liver samples with increasing grades of fibrosis and cirrhosis (30). Aetiology specific differences have also been observed. Mazza and colleagues report increased collagen 10A, 5A, fibulin 5, and fibronectin in cirrhotic liver from patients with HCC (31). Whereas, advanced ALD is associated with increased deposition of collagen type 1, upregulated synthesis of osteopontin (OPN) and increased synthesis of cellular fibronectin (cFN). To date, ECM accumulation in NASH has not specifically characterized.

**Table 1:** Disease-specific changes in the extracellular matrix (ECM).

Disease	Disease stage-specific ECM composition and organisation
**ALD**	Advanced ALD associated with: - Increased collagen type 1 (24) - Upregulated OPN synthesis, correlating with disease severity (25) - Increased cFN synthesis (26) ECM organization - Collagen deposition around hepatic sinusoids (24)
**NASH**	During progressive NASH - General accumulation of ECM proteins (27) ECM organization - Initially perisinusoidal and pericellular. Chicken wire pattern (27)
**HCV**	Progressive HCV - General increase in the relative abundance of elastin, proteoglycan 2, and collagens 10A1, 16A1, and 12A1 with progressive fibrosis and down regulation of fibrinogen, tenascin-X and biglycan (28) Early HCV - Progressive accumulation of collagen I and III (28) Advanced HCV - Pronounced elastin overexpression (28) - Downregulated fibrinogen, tenascin X and biglycan (28) ECM organization - Development of fibrotic septa that bridge portal areas to central veins or adjacent portal areas (29)

Much of this information is available thanks to ongoing advances in proteomics analysis and the development of online platforms, such as MatrisomeDB (32), which allows comparison between healthy liver ECM and diseased liver ECM from different aetiologies. However, currently available proteomics data on human liver ECM is relatively limited, and it is difficult to draw conclusions on which ECM proteins are upregulated or downregulated in different liver diseases and during disease progression and understand their biological context.

## Inflammation in Chronic Liver Disease

Inflammation is central to the pathogenesis and progression of CLD (33). In general, pro-inflammatory immune responses are thought to drive progressive CLD through bystander cell damage and activation of fibrogenic and hepatocarcinogenic pathways. Anti-inflammatory responses may counter these injurious pro-inflammatory responses and slow pathogenesis, but excessive anti-inflammatory responses may inadvertently impair immune-mediated wound healing and induce a state of immune insufficiency leading to a vulnerability to bacterial infection and increase the risk of hepatic decompenzation, as seen in patients with liver cirrhosis (34). A number of immune cells contribute to liver inflammation in CLD, including resident macrophages (KCs), infiltrating monocyte-derived macrophages, T cells, dendritic cell (DC)s and neutrophils. These immune cells, and especially macrophages, activate HSCs, and chronic activation drives progressive fibrosis and ECM remodeling (35, 36). The causative mechanisms responsible for the development of non-resolving and chronic inflammation varies dependant on disease aetiology and is appraised in detail in these reviews (35, 37). For example, ALD inflammation is primarily driven through loss of barrier integrity in the gut and excessive translocation of gut-derived bacterial pathogen-associated molecular pattern (PAMP)s, such as lipopolysaccharide (LPS), and bacterial PAMPs (22) which trigger release of pro-inflammatory cytokines from KCs (22). This in turn drives recruitment of infiltrating neutrophils, a prominent feature of alcoholic hepatitis (37). Inflammation in NAFLD is initially thought to be driven by lipotoxicity (accumulation of toxic lipids in hepatocytes). Lipotoxicity promotes chronic inflammation through activation of KCs and recruitment of infiltrating monocytes, resulting in induction of pro-inflammatory intracellular pathways, such as transforming growth factor (TGF)-β1 (38, 39) promoting HSC activation and driving progressive fibrosis. However, in later stages increased bacterial translocation also contributes to hepatic and systemic inflammation. The concomitant presence of infiltrating monocyte-derived macrophages and KC activation is not exclusive to NASH and is seen in progressive liver fibrosis underlying other CLDs, including HBV and HCV, where viral infection is followed by rapid recruitment of pro-inflammatory monocytes and T-cells into the liver (40). Chronic infection with HBV and HCV drives both inflammatory and anti-viral suppressive immune responses and generally leads to progressive fibrosis and ECM remodeling.

Although multiple mediators of inflammation have been described in CLD, including cytokines, chemokines, DAMPs, and PAMPs, the immunomodulatory role of the ECM has largely been ignored and in particular the ability of the ECM network to modulate immune responses through mechanotransduction, and changes in organ stiffness is not well described.

## ECM Stiffness and Inflammation

Remodeling and accumulation of ECM during fibrosis results in gradual liver stiffening. This is not only one of the results of the fibrogenic process, but also an important promoter of the progression of liver fibrosis itself. Central to the mechanosensing regulation of hepatic cells are the Hippo-related transcriptional coactivators Yes-associated protein/transcriptional coactivator with PDZ-binding motif (YAP/TAZ) (41). In patients with organ fibrosis, a feed-forward cycle of matrix stiffness, myofibroblast activation and proliferation, and ECM accumulation perpetuates fibrosis progression (42), and YAP/TAZ mechanosignaling represents a core pathway of this cycle. The regulation and biological effects of YAP/TAZ signaling are cell type specific (43). In liver fibrosis, YAP is activated in HSCs in response to matrix stiffening, ultimately resulting in the translocation of YAP/TAZ from the cytoplasm to the nucleus and inducing the expression of profibrotic genes and increasing expression of alpha- smooth muscle actin (α-SMA) and excessive ECM deposition (44, 45). In hepatocytes, YAP/TAZ activity was increased in chronic liver injury in mice, and YAP-expressing hepatocytes activated the expression of pro-fibrogenic proteins (collagen I, TIMP-1, platelet-derived growth factor (PDGF) -c, TGF-β2) and pro-inflammatory factors (tumor necrosis factor (TNF)-α, Interleukin (IL) -1β), and stimulated the expansion of myofibroblasts and macrophages (46). This was confirmed in experiments with YAP and YAP/TAZ knockout (KO) animals, where hepatocytes exhibited limited myofibroblast expansion, inflammation, and decreased fibrosis after CCl_4_ injury.

Macrophages can sense changes in stiffness through mechano-transduction signaling and respond by regulating Toll-like receptor (TLR)-mediated inflammation. Macrophages grown on softer substrates induce stronger TLR4 and TLR9 signaling and release higher amounts of the strong pro-inflammatory cytokine TNF-α (47). These events are clearly influenced by growth surface stiffness, as inhibition of mechanotransduction signaling enhances TLR signaling, which increases TNF-α production and prolongs activation of TLR downstream kinases p38 and extracellular signal-regulated kinase (ERK)1/2. ECM deposition that occurs in hepatic fibrosis drastically increases tissue stiffness, indicating that these TLR-mediated events could be involved in modulating inflammatory microenvironments in CLD.

Active ECM remodeling and accumulation underpins liver stiffening in fibrosis, and results in release and/or activation of a range bioactive molecules harboured in the ECM. These molecules can directly and indirectly modulate immune responses during CLD.

## ECM Protein Association With Growth Factors, Cytokines, and Chemokines

ECM proteins anchor a number of growth factors, cytokines, and chemokines, creating a reservoir of bio-active, immunomodulatory molecules. The release and availability of these molecules is regulated by ECM proteins in response to injury, wound-healing, and tissue regeneration, creating a local inflammatory milieu which can drive recruitment of immune cells. In turn, recruited immune cells can actively remodel the ECM network, both by secreting and digesting ECM proteins, further contributing to the release of ECM-bound molecules.

One such ECM-bound molecule, is TGF-β1. TGF-β1 is stored in the ECM and responds to perturbations in the microenvironment to ensure ECM homeostasis (48, 49). This is coordinated through a tightly controlled mechanism of deposition into and release from the ECM. Secreted TGF-β1 is held inactive through its association with a complex of proteins termed the large latency complex (LLC). The LLC, in turn, is anchored to fibrillar ECM proteins (fibronectin, fibrillins, fibulins) (48) by latent- TGF-β- binding proteins (LTBP)s, creating a reservoir of inactive TGF-β1 within the ECM. Active TGF-β1 can be released from the LLC by either proteolytic degradation or mechanical deformation of the complex. Cells bearing αν integrins bind the latency associated protein (LAP) within the ECM- anchored LLC. This generates a pulling force causing a conformational change in the LLC that frees active TGF-β1 (50, 51, 52). The integrin-dependent mechanical release of TGF-β1, explains why latent TGF-β1 can only be activated if bound to the ECM. Mechanical resistance by the ECM ensures that when force is applied to LAP, it will produce the conformational change needed to free TGF-β1. Consequently, increasing ECM stiffness has a direct effect on TGF-β1 activation. Disorganized ECM, typical of remodeling tissues, does not provide enough resistance to induce release of TGF-β1, while stiffer ECM, typical of advanced fibrosis, is expected to ensure sufficient resistance for latent TGF-β1 activation by cell pulling (48).

Most hepatic cells are sensitive to TGF-β1 and in fibrogenesis excessive TGF-β1 activates HSCs inducing their transdifferentiation into myofibroblasts, which further deposit ECM proteins. TGF-β1 also contributes to ECM deposition by amplifying hepatocyte death (53). Furthermore, timely release of TGF-β1 in response to changes in ECM stiffness during liver fibrosis can drive both pro-inflammatory and inhibitory immune responses. For example, TGF-β1 is a key mediator of the terminal differentiation of regulatory T cells (Tregs), important negative regulators of the inflammatory process in liver fibrosis. Studies have also shown that TGF-β1 is immunosuppressive through induction of macrophages, impairment of DC and natural killer (NK) cell activity (52, 54, 55, 56).

One way in which TGF-β1 drives immune responses is thorough the establishment of paracrine senescence in hepatic cells. This describes the transmission of senescence properties from senescent cells to otherwise normal cells in acute and chronic liver injuries (57). Senescence is considered as a multistep, dynamic cellular process in response to endogenous and exogenous stress, such as oncogene activation, DNA damage or other stress-mediated signals, which induces cells to enter a permanent cell cycle arrest. Senescent cells with cell cycle arrest undergo chromatin remodeling, present senescence-associated secretory phenotype (SASP), change morphology and gain other characteristics of a complete senescence phenotype. In the liver, cellular senescence may cause loss of regenerative capability affecting the function and tissue renewal (58). The SASP is a dynamic pro-inflammatory response that activates and reinforces the senescent phenotype in the surrounding cells and modulates fibrosis (57). Most senescent cells express multiple cytokines such as IL-8, chemokine ligand (CCL)-2 (monocyte chemoattractant protein (MCP)-1), CCL7 (MCP-3), CCL8 (MCP-2), CCL13 (MCP-4), CCL3, etc (59, 60). The most characterized SASP components include multiple pro-inflammatory cytokines: IL-1β, IL-6 and TGF-β1. Thus, TGF-β1 is a critical element for the establishment of paracrine senescence through the SASP. TGF-β1 induces and maintains paracrine senescence through a mechanism that generates reactive oxygen species (ROS) and DNA damage response (DDR) (61).

Senescence has been reported in hepatocytes in different liver diseases such as cirrhosis (62, 63) in cholangiocytes during primary biliary cirrhosis (PBC) and primary sclerosing cholangitis (PSC) (64, 65, 66); and in HSCs in fibrosis (67). Specifically, acute liver injury was shown to induce senescence predominantly in hepatocytes *in vivo*, and this was amplified and transmitted between hepatocytes in a feedback loop TGF-β1-dependent produced by macrophages (68). In a model of biliary disease, senescence was specifically induced in cholangiocytes, with evident alterations in the cellular and signaling microenvironment, recruitment of myofibroblasts and macrophages causing collagen deposition, TGF-β1 production and induction of senescence in surrounding cholangiocytes and hepatocytes. The induction of senescence in cholangiocytes also results in the establishment of paracrine senescence in the liver parenchyma through TGF-β1-dependent mechanisms (64, 69).

Numerous ECM proteins in the liver can interact with TGF-β1 and regulate its local availability and activation, which in turn can modulate immune cell responses. For example, Thrombospondin-1 (TSP-1) is an endogenous activator of TGF-β1 during tissue repair and remodeling, causing release of active TGF-β1 from the LLC through a non-proteolytic mechanism (52, 70, 71). The ability to regulate local TGF-β1 release provides TSP-1 certain immunomodulatory properties (49, 72). TSP-1 null mice have a lower TGF-β1 activity that reduces T helper cell (Th)17 and IL-17 levels (73, 74). TSP-1 is implicated in the activation of TGF-β1 by human NK cells (75) and also seems to be responsible for CD36-dependent activation of TGF-β2 by murine antigen presenting cells, a growth factor important for induction of Foxp3^+^Tregs (76).

Biglycan and decorin have been shown to inhibit TGF-β1 activity. Biglycan inhibits the activity of TGF-β1 *in vitro (*77) and *in vivo*, as demonstrated by experiments in biglycan-deficient mice that showed elevated levels of active TGF-β1 in plasma (78), and decorin is reported to bind to and inhibit multiple growth factors and growth factor receptors including TGF-β family members (79).

In liver fibrosis, TGF-β1 availability is regulated by fibronectin (FN), and directly affects collagen fibrillogenesis in response to liver injury (80). Similarly, increasing local availability of TGF-β1 by tenascin-C promotes fibrogenesis through activation of fibroblasts to produce ECM (81). Importantly, Tenascin-C is upregulated in patients with cirrhosis, HCC and chronic hepatitis C (82, 83, 84, 85). Extracellular matrix protein 1 (ECM-1) is reported to downregulate TGF-β1 activity. ECM-1 is produced in the liver mainly by hepatocytes and is downregulated upon liver damage. This event directly induces release of activated TGF-β1 from its latent complex. Furthermore, ECM-1 inhibits latent TGF-β1 activation during homeostasis preventing spontaneous fibrogenesis. In fact, ECM-1 supplementation can reverse fibrosis when ECM-1-knockout mice are exposed to liver damage (86).

Other ECM proteins which are reported to bind TGF-β include fibrillin-1, plasma fibronectin (pFN) (48), heparan sulphate (HS), and agrin which can bind to both TGF-β1 and TGF-β2 (87) and regulate local gradients and growth factor activity (88, 89).

Many ECM proteins bind a range of growth factors and are summarized in **Table 2**. Some examples are represented by biglycan, which interacts with TNF-β (103), bone morphogenetic proteins (BMPs) BMP-2, BMP-4, BMP-6 (104, 105), and Wnt-1-induced secreted protein 1 (WISP1) (106). Decorin sequesters PDGF before it can bind to receptors on target cells, thus blocking its activity (107). Similarly, Decorin binds to and neutralizes connective tissue growth factor (CTGF) (108), Low density lipoprotein receptor related protein 1 (LRP1) and WSP1 (106). Fibrillin-1 binds to BMPs, integrins responsible for cell-matrix communication, and other growth and differentiation factors (100–102, 109). pFN binds to vascular endothelial growth factor (VEGF), BMP-1, hepatocyte growth factor (HGF) fibroblast growth factor (FGF)-2, PDGF, and latent TGF-β1 (48). Finally, tenascin-C binds to the FGF family, VEGF, PDGF, and insulin growth factor-binding proteins (IGF-BPs) (95, 96). A detailed account of the specific interactions can be found in this review (4).

**Table 2:** Growth factors, chemokines, and cytokines that bind to specific extracellular matrix (ECM) proteins.

ECM molecule	Bioactive molecule
**Thrombospondin-1**	**Growth factors:** TGF-β1 (52, 70, 71)
**Agrin**	**Growth factors:** TGF-β1, TGF-β2 (87)
**Heparan Sulphate**	**Growth factors:** TGF-β1, TGF-β2, FGF (4, 88, 89) **Chemokines:** CXCL8 (90)
**Heparin** **(Highly sulphated form of HS)**	**Growth factors:** FGF, VEGF, PDGF, HGF (4) **Cytokines:** IL-4 (91) IFNγ (92) RANTES, IL-7 (93, 94)
**Tenascin-C**	**Growth factors:** TGF-β family, FGF family, VEGF, PDGF, IGF-BPs (95, 96) **Cytokines:** CCL21 (97)
**Plasma Fibronectin**	**Growth factors:** VEGF, BMP-1, HGF, FGF-2, PDGF, TGF-β1 (48)
**Fibronectin**	**Cytokines:** IL-7 (94), IL-2 (98), TNF-α (99)
**Laminin**	**Cytokines:** IL-7 (94), IL-2 (98), TNF-α (99)
**Fibrillin-1**	**Growth factors:** TGF-β1, BMPs (100, 101, 102),
Proteins that have been shown to inhibit GF activation	
**Biglycan**	**Growth factors:** TGF-β1 (77, 78), TNF-β (103), BMP-2, BMP-4, BMP-6 (104, 105), WISP1 (106)
**ECM-1**	**Growth factors:** TGF-β1 (86)
**Decorin**	**Growth factors:** TGF-β1, PDGF (79), CTGF (107), WISP1, LRP1 (108)

The ECM also harbours a large number of cytokines and chemokines secreted, for example, by immune cells infiltrating to sites of damage or infection. Many ECM proteins have affinity for these molecules creating a chemoattractant and immune-modulatory gradient further attracting and activating incoming immune cells. For example, heparan sulphate binds IL-4 (91),which primarily acts on B cells; interferon (IFN)γ (92), which acts on monocytes, T cells, DCs, and NK cells; Regulated on Activation Normal T cell Expressed and Secreted (RANTES) (93), which acts on memory T cells, eosinophils, basophils, NK cells, and DCs; and IL-7 (94). Similarly, IL-7 and IL-2 bind fibronectin (94), laminin, and collagen IV; while TNF-α (98), which acts on T cells, B cells, monocytes, endothelial cells and fibroblasts binds with FN (110) and laminin (99). Finally, tenascin-C binds to CCL21 enforcing an immune-suppressive lymphoid stroma, as shown in a model of oral squamous cell carcinoma (97). By inducing CCL21 and binding to it, tenascin-C instructed lymphoid stroma to recruit T regulatory cells and express high levels of anti-inflammatory cytokines.

## Immunomodulatory Properties of the ECM

In addition to influencing immune responses through the release and modulation of bioactive molecules, ECM components can directly modulate immune responses. The prevalence, topological organization and molecular structure of ECM proteins changes during tissue remodeling and wound repair, as well as during disease development, progression and inflammation (111, 112). Changes in the ECM occur in both healthy and diseased tissues, and matrix molecules are able to create microenvironmental niches that affect intracellular signaling pathways critical for the regulation of local cells and immune cells. Many ECM proteins harbour bioactive domains which act as direct ligands on a large number of immune cell receptors (113, 114). ECM ligand-immune cell interactions drive both pro- and anti-inflammatory immune responses and are involved in maintaining homeostasis but also in establishing pathways of pathogenesis. For example, collagen is a high affinity ligand for leukocyte associated immune receptor (LAIR)-1 expressed on most immune cells including T cells, B cell, NK cells, monocytes, macrophages, monocyte-derived DCs, mast cells, eosinophils, and basophils (115). Collagen-LAIR-1 association promotes immune suppressive phenotype essentially holding the immune response in check and contributing to homeostasis. Similarly, differential expression of LAIR-1 by immune cells or interruption of collagen-LAIR-1 binding by the action of soluble receptor LAIR-2 (115), may be involved in driving disease processes. Indeed, Martinez-Esparza et al., found a reduction of LAIR-1 expression on macrophages in the liver of patients with liver cirrhosis but an increase LAIR-1 expression in circulating monocytes in the blood of cirrhosis patients (116). The authors suggest that upregulation of collagen in the cirrhotic liver may serve to inhibit LAIR-1 expression on liver macrophages, suggesting a role for collagen-LAIR-1 interaction in liver cirrhosis.

**Figure 1:**
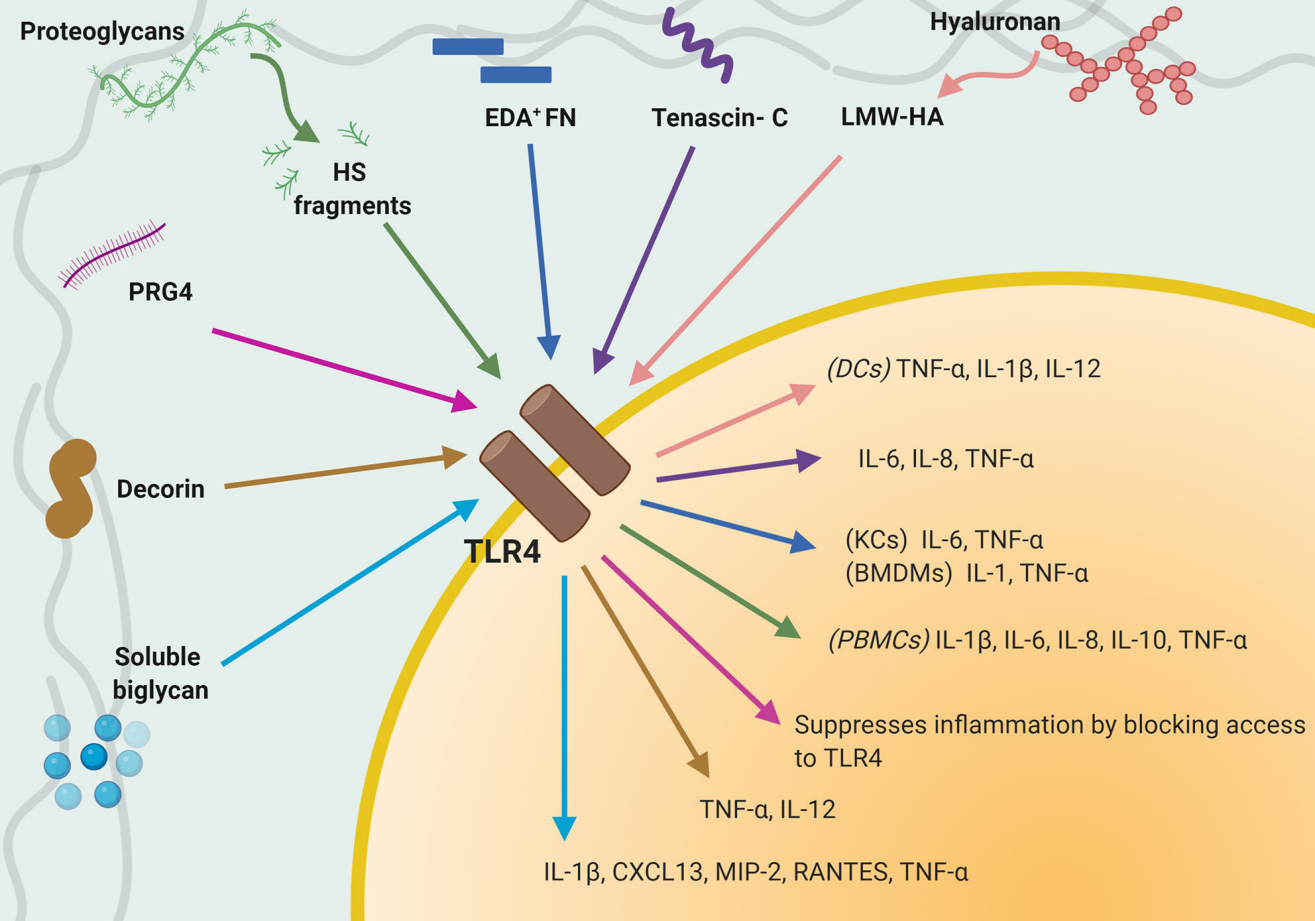
Interaction between TLR4 expressed on immune cells and extracellular matrix (ECM) proteins/ECM-damage-associated molecular patterns (DAMPs). Schematic that summaries known interactions between TLR4 and individual ECM-DAMPs or ECM proteins and consequent immune responses.

In addition, newly synthetized or fragmented ECM proteins, generated upon remodeling or during injury, are recognized by immune cells as DAMPs, or in this case, ECM-DAMPs (117, 118). ECM-DAMPs engage directly with multiple immune cells, including macrophages (119–122), monocytes (123), dendritic cells, and T cells (124), as well as with endothelial cells (3, 41), and modulate a wide range of pro- and anti-inflammatory responses, which will be discussed in the following sections. These endogenous glycoproteins, peptides, proteoglycans and GAGs support infiltrating immune cells and proliferating tissue resident cells to orchestrate sterile inflammation, for example by modulating TGF-β and IL-1β signaling (117, 125–128). ECM-DAMPs engage with multiple pattern recognition receptors (PRRs), which recognize and induce activation of cells of the innate and adaptive immune system such as monocytes, macrophages and T cells, and non-immune cells like endothelial cells. Among the PRRs identified as able to bind ECM-DAMPs, TLR2, and TLR4 play a preponderant role as effectors of their immunomodulatory properties (129). A summary of interactions between TLR4 on immune cells and ECM proteins/ECM-DAMPs and consequent immune response is shown in **Figure 1**. ECM-DAMPs promote both pro- and anti-inflammatory immune responses, suggesting that the matrix proteins are actively involved in fine tuning the inflammatory processes (117, 130–132).

This evidence demonstrates that the ECM is a biologically active participant in perpetuating inflammation and suggests an important role in the progression of liver fibrosis and inflammatory disease. However, there is a lack of knowledge of specific ECM-immune interactions involved in CLD. Exploring the role of immunomodulatory ECM proteins in CLD could be key to understanding the inflammatory responses upon liver injury, providing new biomarkers to identify disease stage, predict disease progression, and develop new anti-inflammatory or immunomodulatory drugs. Some of these proteins have been studied for their immunomodulatory properties in the healthy liver, in liver cancer and in CLD, for example the role of periostin and TSP-1 in NAFLD and NASH. Other ECM components have been marginally studied in the liver but have been identified as key players in other inflammatory environments, such as the recruitment and induction of cytokine synthesis in macrophages by decorin and versican. Individual ECM molecules and their known pro- and anti-inflammatory role in inflammatory diseases are reported in the next sections. Some ECM components have been briefly studied in the liver but have been identified as key players in other inflammatory environments.

**Figure 2:**
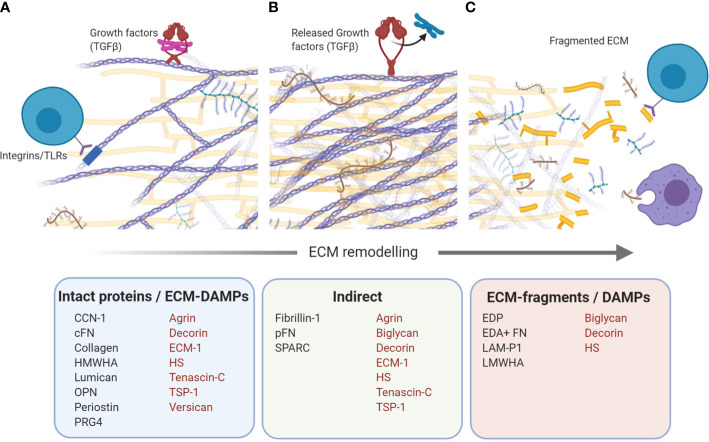
Immunomodulatory extracellular matrix (ECM) proteins. Schematic that shows ECM proteins with direct and indirect modulation of immune responses. **(A)** Intact proteins/ECM-damage-associated molecular patterns (DAMPs). Bioactive domains on numerous ECM proteins act as direct ligands for a wide number of immune cell receptors, including toll-like receptors (TLRs) and integrins, and drive a range of inflammatory and immune-suppressive responses. **(B)** Indirect. Numerous ECM proteins can bind growth factors, harbouring them in the ECM. Growth factors can be released upon ECM remodeling, creating a local inflammatory milieu which drives recruitment and polarization of infiltrating and resident immune cells. **(C)** ECM-fragments/DAMPs. Active remodeling of the ECM during fibrosis, results in the generation of ECM protein fragments which can be recognized by immune cells as DAMPs. Proteins with more than one mechanism of immunomodulation are indicated in red. Schematic was created using BioRender (https://app.biorender.com).

Over 150 ECM proteins have been identified in the human liver (9, 30). In this review we highlight 20 liver ECM proteins, selected for their implication in CLD and that have been shown to modulate immune responses. For clarity, we have grouped these proteins as “pro-inflammatory” – those with predominantly pro-inflammatory actions (versican, TSP-1, agrin, cysteine-rich protein 61, lumican, periostin, heparan sulphate, low molecular weight-hyaluronic acid, biglycan, elastin, and secreted protein, acidic and rich in cysteine); “anti-inflammatory” - those with predominantly anti-inflammatory actions (ECM-1, high molecular weight-hyaluronic acid and proteoglycan-4); and “dual acting”- those which have distinct pro- and anti-inflammatory properties (tenascins, OPN, collagen, decorin, and fibronectin). Further, where possible, proteins are organized as those which drive immune responses through direct ligand-immune cell interactions, followed by proteins which are recognized as ECM-DAMPs (**Figure 2**). Each protein is discussed for its specific immune-modulatory properties and the known implications in CLD. This is summarized in **Table 3**.

**Table 3:** Specific immune-modulatory properties of individual ECM proteins and known implications in CLDs.

Protein	Receptors	Immune activity	In CLD
**Pro-inflammatory**			
**Versican**	TLR2, TLR6, CD14 (133)	**Direct Ligand: Immune cell interactions** - Induces production of IL-6 and TNF-α in bone marrow-derived macrophages through TLR2/TLR6 or CD14 interaction (133) - Influences M1/M2 polarization of macrophages (134)	- Overexpressed in liver fibrosis from HCV infection (135)
**TSP-1**	CD36 (136), CD47 (137,138), Integrins (139)	**Direct Ligand: Immune cell interactions** - CD36:TSP-1 binding activates macrophages (136)	- Minimally expressed in normal liver (140) - Upregulated in liver fibrosis, alcohol cirrhosis, NASH-cirrhosis and HCV infection (140, 141) - Involved in steatosis and steatohepatitis in NAFLD (139) - Associated with fat accumulation, inflammation and fibrogenesis in NAFLD *in vitro* (142)
**Agrin**		**Direct Ligand: Immune cell interactions** - Orchestrates the interaction between T cells and their antigen-presenting partners (143) - Survival factor for monocytes (144)	- Normally expressed at low level in portal arteries and biliary compartment (145, 146) - Upregulated in cirrhosis and HCC (145)
**CCN1**	α_M_β_2_, syndecan-4 (147,148)	**Direct Ligand: Immune cell interactions** - Induces macrophage adhesion and activation through α_M_β_2_ integrin and syndecan-4, leading to synthesis of pro-inflammatory M1 macrophage cytokines and chemokines including TNF-α, IL-1α, IL-1β, IL-6, IL-12b, IFN-γ, MCP-1, MCP-3, MIP-1α, MIP-1β, and IP-10 (147,148)	- Induces monocyte infiltration in NAFLD inducing severe hepatic inflammation (149) - Increases senescence in HSC and portal myofibroblasts reducing liver fibrosis (150,151) - Stimulates expansion of MDSCs (150, 152, 153, 154),
**Lumican**	CD14 (155)	**Direct Ligand: Immune cell interactions** - Involved in recruitment of neutrophils and macrophages (156,157) - Its deficiency affects resolution of inflammation (158,159) - Interacts with CD14 to present LPS (155)	- Increased expression in rat models of fibrosis (160) and in patients with NAFLD, NASH, HCV- and HBV-associated fibrosis (161, 162, 163, 164), - Correlates with progression of NAFLD to NASH (162)
**Periostin**	α_v_β_1_ (165,166)α_v_β_3_ (166,167), α_v_β_5_ (106,167), α_6_β_4_ (168), α_M_β_2_ (169)	**Direct Ligand: Immune cell interactions** - Upregulated in various inflammatory diseases (170,171,172, 173, 174), - In asthma, it correlates with Th2-high phenotype with high expression of IL-13 and IL-5 (175,176) - Amplifies Th2 immune response in allergic skin inflammation and facilitates adhesion of eosinophils to the ECM (177)	- Upregulated in liver fibrosis in a TGF-β1-dependent manner (178) - Involved in macrophage infiltration and liver fibrosis development (111) - Increased serum level correlates with higher levels of TGF-β1 and TGF-β2 in patients with acute and chronic hepatitis (111) - Implicated in NAFLD progression to NASH (179,180)
**Heparan sulphate**	L-Selectin (181), CD14 (182), TLR1, TLR2, TLR4, TLR6 (183–186),	**Direct Ligand: Immune cell interactions** - Modulates the innate immune response by facilitating activation of CD14 and TLR4 by LPS (182) ECM-DAMP - ECM-DAMP: HS fragments are reported to induce pro-inflammatory cytokine release from PBMCs *via* TLR4 (IL-8, IL-6, IL-1β, TNF, IL-10) (187)	- Increased expression in cholestatic liver (188) - Facilitates viral particle binding in both HBV and HCV (189,190)
**LMW-HA**	TLR2, TLR4, CD44 (119,120,122,191–193)	**Direct Ligand: Immune cell interactions** - Generated during inflammation and tissue injury by endogenous and bacterial hyaluronidases and by mechanical forces and oxidative stress (194,195) - Activates pro-inflammatory response in macrophages (119, 121, 122), dendritic cells *via* TLR4 and endothelial cells (191) - Stimulates macrophages to produce IL-8, IL-12, MIP-1α, MIP-1β, and MCP-1 (119, 121, 122) - Stimulates DCs and endothelial cells to produce IL-1β, TNF-α, and IL-12 (191) ECM-DAMP - LMW-HA is considered an ECM-DAMP known to activate the inflammasome and subsequently caspase-1 (126). The activation of the inflammasome is induced by lysosomal digestion of LMW-HA into fragments upon internalization by macrophages (196)	- Hyaluronic acid is upregulated in liver connective tissue of patients with steatosis, steatohepatitis, cirrhosis, and HCC (197)
**Biglycan**	TLR2, TLR4 (198)	**Direct Ligand: Immune cell interactions** - Proteolysis of ECM caused by stress or tissue damage releases soluble biglycan - Binds to TLR2 and TLR4 mediating innate immunity and leading to activation of p38, Erk and NF-κB and finally to a pronounced expression of TNF-α and MIP-2 (198), as well as IL-1β (199,200), CXCL13 (200), MIP-1α, MCP1 and RANTES (201) ECM-DAMP - Cleavage of biglycan by proteases can lead to generation of fragments; some of which are able to bind to TLR2/4	- Biglycan-derived fragments are found in serum of rat models of liver fibrosis (202)
**EDPs**	ERC (203)	**ECM-DAMP** - Stimulate T cells to release IFN-γ (124) - Drives polarization toward a Th-1 response (204) - Modulates the expression of proinflammatory cytokines TNF-α, IL-1β and IL-6 in monocytes (123)	- EDP production during aging plays an important role in the development of NASH (205)
**SPARC**		- No direct evidence of interaction with immune cells, however, induces inflammatory cell recruitment in kidney, lung and skin fibrogenesis (206,207)	- Constitutively expressed in the normal liver (208) and is upregulated in activated HSCs (209), in rat and human cirrhotic liver (208,210,211) and in HCC (212, 213, 214),
**Anit-inflammatory**			
**ECM-1**	CD122, αv integrins (215,216)	**Direct Ligand: Immune cell interactions** - Inhibits Th17 cell differentiation *via* CD122 (216) - Can interact with αv integrin on DC cells and block the αv integrin-mediated activation of latent TGF-﻿β (216) - Involved in Follicular helper T cell differentiation and antibody response, upstream of B cell activation (217) - Stabilizes TGF-﻿β in its inactive form (86)	- Regulates liver tissue homeostasis and inhibits latent TGF-β1 activation (86) - Downregulated upon liver damage, resulting in activation of TGF-β1 (86) - Inversely correlated with severity of liver fibrosis for patients with ALD and HBV (86)
**HMW-HA**	TLR2 (192) CD44 (125,218)	**Direct Ligand: Immune cell interactions** - Inhibits TLR2 signalling (192) - Inhibits phagocytosis in monocytes (219) - Limits antigen-antibody interactions (220) - Promotes Foxp3 expression in Tregs cells *via* CD44 and death of activated T cells (118, 221) - Promotes induction of CD4^+^Foxp3^-^ TR1 cells (218)	
**PRG4**	TLR2, TLR4, CD44 (222–225),	**Direct Ligand: Immune cell interactions** - Promotes anti-inflammatory response in osteoarthritis and rheumatoid arthritis. Interacts with fibroblasts through TLR2, TLR4 and CD44, suppressing proliferation and production of pro-inflammatory molecules (222–225),	- Highly expressed in the liver and increases with high fat diet (225) - PRG4-KO animals in high fat diet show reduced hepatic steatosis and inflammation (225)
**Dual-Activity**	
**Tenascin C**	EGFR (112) TLR4 (82, 226–230), α_9_β_1_ and α_v_β_3_ (226)	**Direct Ligand: Immune cell interactions** - Upregulated in response to LPS, induces production of IL-6, IL-8 and TNF by inflammatory cells (macrophages, DCs) *via* TLR4 (82, 226–232), - Involved in Th17 differentiation (233) - Drives chronic inflammation in autoimmune, neurological, metabolic and fibrotic diseases (96, 112) - Suppresses T-cell activation (232, 234–237), Modulates sterile inflammation during tissue repair and contributes to chronic inflammation *via* TLR4, α_9_β_1_ and α_v_β_3_ (226)	- Upregulated in rat models of liver fibrosis (238) and in patients with cirrhosis, HCC and chronic hepatitis C (83–85), - Implicated in activation of HSCs and lymphocyte activation and infiltration in immune-mediated hepatitis (239)
**OPN**	CD44, α_v_β_3_ (218, 240)	**Direct Ligand: Immune cell interactions** Pro-inflammatory - Inhibits IL-10 production by Tregs cells through interaction with CD44 (218) - Chemoattractant for macrophages and neutrophils (241,242) Anti-inflammatory - Reported to reduce production of IL-6, TNF-α, and IL-1β by macrophages (243)	- Associated with alcoholic cirrhosis, ALD, acute liver failure, NAFLD, HBV- and HCV-fibrosis and HCC (24, 244–251), - Induces reduction of IL-6, TNFα, IL-1β and toxic iNOS by macrophages in liver injury (252) - Correlates with severity of liver fibrosis (241) - Activates HSCs (253) and correlates with TGF-β1 expression in CLD (248, 254)
**Collagen**	LAIR-1, LAIR-2 (255, 256, 257), α_1_β_1_, α_2_β_1_, α_10_β_1_, α_11_β_1_, OSCAR (258), GPR56 (259, 260, 261),	**Direct Ligand: Immune cell interactions** Pro-inflammatory - Mediates pro-inflammatory signals through LAIR-2 on PBMCs, monocyte and T cell lines (118, 262) Anti-inflammatory - Suppressive immune activity through LAIR-1 on NK cells, T cells, B cells, monocyte/macrophages, dendritic cells, basophils, mast cells (263)	- Increased deposition of collagen in liver fibrosis and cirrhosis - Higher expression of LAIR-1 by peripheral blood monocytes in cirrhotic patients but lower expression in hepatic tissue (116)
**Decorin**	TLR2, TLR4 (264, 265), α_2_β_1_ (266)	**Direct Ligand: Immune cell interactions** Anti-inflammatory - Suppresses TGF-β activity (79, 201, 267, 268) - Promotes acute inflammatory reaction in peritoneal macrophages *via* TLR2 and TLR4, stimulates TNF-α, IL-12 expression, and inhibition of IL-10 (265) ECM-DAMP Pro-inflammatory - Fragments which can act as ECM-DAMPs through TLR2 and TLR4 driving pro-inflammatory and pro-fibrotic activity (79,269,117)	- Upregulated during NASH progression (270)
**Fibronectin**	TLR4 (271,272)	**Direct Ligand: Immune cell interactions** Pro-inflammatory - THP-1 monocyte/macrophages show increased MMP-9 production in response to recombinant FN- EDA and EDB *via* TLR4 signalling (272) - KCs stimulated by exogenous cFN produced the pro-inflammatory cytokines, TNF-α and IL-6, as well as pro-fibrogenic factors, MMP-2 and TIMP-2 (273) ECM-DAMP Pro-inflammatory - EDA containing FN fragments activate macrophages *via* TLR4, in a MD-2 co-expression dependent manner (272) Anti-inflammatory - Stimulate production of IL-10 and IL-13 in human fibroblasts *via* TLR4 (271)	- Early matrix molecule in fibrosis, it links TLR4 activation of HSCs and angiogenesis (274) - Biomarker for early stage ALD (275) - Induces TNF-α and IL-6 expression in hepatocytes and other inflammatory cytokines by KCs (273,276)

Other notable ECM proteins that are not reported to modulate the immune responses, but which are involved in CLDs: Laminin. Despite the lack of evidence for a direct effect on the immune system, laminin fragment P1 (LAM-P1) serum level, generated by pepsin digestion (277), was found to correlate with the degree of fibrosis from perivenular fibrosis to septal fibrosis to cirrhosis, and with inflammation, in patients with ALD (278).

## ECM Proteins Which Drive Pro-Inflammatory Immune Responses

### Versican

Versican is a chondroitin sulphate proteoglycan; the alternatively spliced GAG chain binding region gives rise to four versican isoforms: V0 (370 kDa), V1 (263 kDa), V2 (180 kDa), and V3 (74 kDa) (201, 279). V1 was shown to induce production of pro-inflammatory cytokines IL-6 and TNF-α in bone marrow-derived macrophages. This pro-inflammatory effect was mediated by interaction of V1 with TLR2 and CD14 on macrophages, but not TLR4. The response through TLR2 required TLR6 as co-receptor (133). Versican is considered to be involved in M1/M2 polarization of macrophages (134) and can also be synthesised and secreted by activated macrophages in inflammatory diseases, such as cardiovascular diseases and fibrosis, and in response to LPS (134, 280–282).

It has been reported that expression of versican increases in cirrhotic liver, where it localizes predominantly in the cytoplasm of hepatocytes, as well as in the perisinusoidal and stromal regions (135, 283). In a study by Ramnath and colleagues, the authors found V0 and V1 isoforms to be elevated in advanced liver fibrosis from either HCV or fatty liver disease, and these patients had elevated serum levels of versican (135). Versican is suggested to play a role in liver fibrosis by activating HSCs in *in vitro* experiments (283). It has been hypothesised that versican could have a pro-inflammatory effect in liver fibrosis as both the intact molecule and products of tissue remodeling by proteases are overexpressed in patients with advanced fibrosis (135).

### Thrombospondin-1

Thrombospondin-1 (TSP-1) is a multi-functional glycoprotein which regulates cell-cell and cell-matrix interactions in tissue homeostasis and tissue repair and is upregulated under pathophysiological conditions (49). TSP-1 is recognized in the extracellular space by CD36 (expressed by endothelial cells, vascular smooth muscle cells, adipocytes and macrophages, among others (136, 284, 285), CD47 [expressed by endothelial cells, epithelial cells, smooth muscle cells, neutrophils, erythrocytes and chondrocytes (137, 138)] and integrins (139). TSP-1 regulates inflammation in a CD36- and CD47- dependent manner, for instance CD36:TSP-1 binding activates bone-marrow derived macrophage (BMDM)s (136).

The pro-inflammatory role of TSP-1 has been described in rheumatoid arthritis, inflammatory joint disease and in liver disease (73, 74, 139–142, 286–291). TSP-1 stimulates adipose tissue F4/80^+^ macrophage recruitment and activation in adipose tissue contributing to inflammation and insulin resistance resulting from high-fat diet-induced obesity (292). In NAFLD, TSP-1 is implicated in stimulating steatosis and steatohepatitis (139), seen by a reduction in lipid accumulation and TNF-α production in TSP-1 deficient mice (139). Similarly, in an *in vitro* model of NAFLD, TSP-1 expression by hepatocytes upon free fatty acid treatment, was associated with fat accumulation, inflammation and fibrogenesis (286). Also, in a partial hepatectomy mouse model, an immediate and transient TSP-1 expression was induced, primarily from endothelial cells and activated HSCs (293). TSP-1 expression was also increased alcohol-treated rats and in mouse models of liver fibrosis induced with CCl_4_ or 3,5- diethoxycarbonyl-1,4-dihydrocollidine (DDC) diet (140).

Although TSP-1 is minimally expressed in the normal liver, hepatocytes and HSCs show deposition of large amounts of TSP-1 in patients with congenital liver fibrosis (291), and TSP-1 is upregulated in liver samples from patients with alcohol cirrhosis and NASH-related cirrhosis (140). In a HCV core transgenic mouse model, as well as in a co-culture model of hepatoma cells and HSCs, HBV core protein was able to induce expression of TSP-1 by hepatic cells and induce activation of TGF-β1 (294).

### Agrin

Agrin is a large, heparin sulphate proteoglycan; its primary function is considered to be the establishment and maintenance of neuromuscular junctions (295), while its role in other tissues and organ microenvironments is less well understood. Agrin was found to orchestrate the formation of the “immunologic synapse” between T cells and their antigen-presenting partners (143), and was identified as a cell-autonomous survival factor for monocytes (144).

In the liver, agrin dramatically increases in the basal membrane of bile ductules and arterial walls in cirrhosis (145). Agrin can be secreted by HSCs activated by PDGF (296). PDGF receptor-inhibition by the multi-kinase inhibitor sorafenib, reduces the hepatocarcinogenesis mediated through agrin secretion and alleviates inflammation and fibrosis. A certain immunomodulatory activity by agrin is suggested by the interactions with T cells, monocytes and its ability to bind to growth factors in the ECM, such as TGF-β1 (87).

### CCN1

The cysteine-rich protein 61, CCN1 or Cyr61, is an ECM-associated matricellular protein that plays a critical role in cell adhesion, migration, proliferation, apoptosis and survival, in both physiological and pathological tissue states (149, 297, 298). The CCN family of small cysteine-rich proteins are implicated in maintenance of normal liver function and pathogenesis of liver diseases (299). CCN1 is particularly expressed during inflammation or tissue repair. It induces macrophage adhesion and activation through integrin α_M_β_2_ and syndecan-4, leading to expression of multiple pro-inflammatory cytokines and chemokines including TNF-α, IL-1α, IL-1β, IL-6, IL-12b, IFN-γ, MCP-1, MCP-3, macrophage inflammatory protein (MIP)-1α, MIP-1β, inflammatory protein 10 (IP-10), and others (147, 148), typical of M1 macrophage phenotype. These effects are a direct result of activation of NF-κB signaling in macrophages by CCN1, and does not involve TLR4 (147).

In a mouse model of NAFLD, CCN1 induced severe hepatic inflammation that was correlated with higher infiltration of monocytes in the liver *via* integrin α_M_β_2_, confirmed by a decreased monocyte infiltration blocking integrin α_M_β_2_ in presence of CCN1 (149). This study indicated that the pro-inflammatory process typical of NAFLD is initiated by CCN1-mediated induction of macrophage infiltration into the liver through direct chemotaxis and recruitment by chemokines. CCN1 is also able to induce expansion of myeloid-derived suppressor cells (MDSCs), a population of regulatory cells that accumulate in the liver (150, 152, 153, 154). CCN1 was secreted at high levels by injured cholangiocytes and hepatocytes in PBC, promoting expansion of MDSCs and contributing to suppression of T-cell proliferation (150).

### Lumican

Lumican is a 40 kDa keratin sulphate proteoglycan involved in assembling collagen fibres and in regulating expression of TGF-β1 and α-SMA (300, 301).

Evidence of lumican participation in inflammatory signaling was seen upon analysing its interaction with the TNF superfamily member 6 that induces secretion of pro-inflammatory cytokines, which are involved in the recruitment of neutrophils and macrophages (156, 157). Furthermore, lumican deficiency reduces infiltration of neutrophils and negatively affects wound healing and resolution of inflammation (158, 159). Decreased sulforylation of lumican side chains stimulates macrophage adhesion and the cellular inflammatory response, suggesting that remodeling of the ECM could produce changes in the structure of lumican establishing the inflammatory microenvironment that promotes collagen deposition during fibrogenesis (302, 155). Interestingly, lumican does not seem to interact with TLR2 and TLR4, but with their adaptor molecule CD14 to present LPS and stimulate the relative immune response (155).

In the liver, lumican expression level increases during fibrosis in rat models of hepatic fibrosis (160) and in patients with NAFLD, NASH, HCV-, and HBV-associated fibrosis (161, 162, 163, 164), where it correlates with progression of fibrosis stage. In liver biopsies from patients with mild and severe NASH, lumican was found increased in sinusoids compared to tissue from obese patients with normal liver or with liver steatosis (162). This data suggests that lumican is expressed differentially across the progressive stages of NAFLD, indicating that this ECM component could be overexpressed at the beginning of the fibrogenic process in NAFLD patients with progressive disease.

### Periostin

Periostin is a 93.3 kDa matricellular protein that interacts with other ECM structures (collagen I, fibronectin, tenascin-C and BMP-1) supporting processes like wound healing and fibrosis (170, 177, 303, 304). The main receptors reported to interact with periostin are integrin α_ν_β_1_ (165, 166), α_ν_β_3_ (166, 167), α_ν_β_5_ (166, 167), α_6_β_4_ (168), and α_M_β_2_ (169).

Studies using animal models and patient samples have highlighted an active role of periostin in the pathobiology of various inflammatory diseases, including fibrosis, arthritis, chronic allergic skin inflammation, atherosclerosis and asthma (177, 171, 305, 306, 172, 173). In patients with asthma, periostin is upregulated and co-localizes with other proteins of the basement membrane such as tenascin-C, collagens I, III, and IV and fibronectin (174, 307), and correlates with “Th2-high” asthma, characterized by high expression of cytokines IL-13 and IL-5 (308, 175).

In allergic skin inflammation, activation of a Th2 immune reaction stimulates release of IL-4 and IL-3 that induce production of periostin by fibroblasts (177). Periostin is then able to activate keratinocytes through interaction with αν integrin to produce pro-inflammatory cytokines including thymic stromal lymphopoietin (TSLP), amplifying the type 2 immune response. Periostin also facilitates adhesion of eosinophils to the ECM (176). Thus, periostin is an important modulator in allergic inflammation by acting on epithelial cells, fibroblasts, eosinophils and possibly other immune cells.

Upregulation of periostin at both RNA and protein level was found in liver tissue of mouse models of liver fibrosis induced using CCl_4_ or BDL (178). Periostin is required for the activation of HSCs by TGF-β1 and promotes HSC migration. Periostin activity on HSC seems to be mediated by interaction with α_ν_β_3_ and α_ν_β_5_ integrins (178). These data were confirmed in studies with periostin-deficient mice, where CCl_4_- and BDL-induced acute and chronic liver fibrosis were drastically attenuated (111). In this experimental approach, periostin expression at liver tissue level and serum level was upregulated in mouse models of acute and chronic hepatic fibrosis, while this effect was abolished in the recovered livers after stopping CCl_4_ administration. Concomitantly, α-SMA and type I collagen upregulation upon liver injury was decreased in periostin-deficient animals. Periostin-deficient mice treated with CCl_4_ for 2 weeks also showed a significantly lower serum level of alanine transaminase (ALT) and aspartate amino transferase (AST), F4/80^+^ hepatic macrophage infiltration and fibrosis development compared to wild type treated mice. Expression of IL-6, IL-1β, and TNF-α was also decreased in the acute hepatic fibrosis model. In these models, TGF-β1 and TGF-β2 RNA expression and serum levels were correlated with presence of periostin: activated HSCs were able to produce periostin upon TGF-β1 stimulation, and periostin-deficient mice with acute and chronic hepatic fibrosis showed a significantly lower expression of TGF-β1 and TGF-β2 compared to wild-type mouse models of fibrosis. This evidence could indicate a potential reciprocal regulatory mechanism between TGF-β and periostin during liver fibrosis, where persistent liver injury and inflammation induces overexpression of periostin *via* TGF-β stimulation, and periostin, in turn, might induce macrophage infiltration and further production of TGF-β. This hypothesis was supported by analyses that indicate an increased serum level of TGF-β1, TGF-β2 and periostin in patients with acute and chronic hepatitis, compared to healthy controls (111). Other studies suggested that periostin expression could be induced by TNF-α and IL-17 (309, 310) contributing to hepatic fibrogenesis.

Periostin is implicated in the progression of NAFLD, where high expression of periostin was identified in high fat diet-fed mice and in obese mice (179, 311). Periostin-knockout mice fed with methionine-choline-deficient diet, which is known to induce NASH, exhibited a markedly lower level of steatosis, inflammation, and fibrosis in the liver compared to wild type animals (180). Similar evidence was reported in clinical research, where serum and liver tissue expression levels of periostin were higher in NAFLD patients in respect to controls (179, 312) and upregulation of periostin depended on the release of pro-inflammatory cytokines in NAFLD patient livers (313).

### Heparan-Sulphate

Heparan sulphate (HS) chains are covalently bound to the core molecules of heparan sulphate proteoglycans (syndecans, perlecans, and glypicans). They are implicated in a variety of developmental and physiological processes as well as diseases including pulmonary, renal, and cystic fibrosis (314, 315, 181, 316), liver cholestasis (188), and viral hepatitis (189, 190).

HS can interact with a range of immune cells and modulate immune responses. It does this directly, by acting as a ligand for L-selectin, a transmembrane glycoprotein expressed on most leukocytes, as well as a binding platform for cytokines and chemokines (such as chemokine (C-X-C) ligand (CXCL) 8) (90) which modulate immune cell recruitment and activation, and is reported to play a role in immune cell infiltration during inflammation (181). HS is able to modulate the innate immune response by facilitating activation of CD14 and TLR4 by LPS (182). In addition to driving direct ligand-immune cell responses, HS fragments can also be recognized by immune cells as ECM-DAMPs. For example, HS fragments are reported to induce the expression of proinflammatory cytokines from human peripheral blood mononuclear cell (PBMC)s, including high levels of IL-8, IL-6, IL-1β, TNF, and IL-10 (187). HS fragments can also be released by heparanases (183) from perlecan (184), syndecans (185), and glypicans (186) and bind to TLR1, TLR2, TLR4, and TLR6. Nevertheless, the presence and activity of these ECM-derived fragments have not been investigated in CLDs.

### Low Molecular Weight-Hyaluronic Acid

The GAG hyaluronic acid (HA) is composed of repeating disaccharides which form chains of variable lengths and molecular weights (118). Different molecular weight HA shows distinct effects on cellular phenotype and behaviour. HA breakdown products are generated during inflammation and tissue injury by endogenous and bacterial hyaluronidases (HYALs) and by mechanical forces and oxidative stress (194, 195). Low molecular weight HA (LMW-HA) (<15 saccharides; <3 kDa) produced during acute and persistent inflammation promotes angiogenesis (317), maturation of antigen-presenting cells (318, 319) and cell migration (320). LMW-HA acts as an endogenous activator of TLR2- and TLR4-promoted immune response and through CD44 (192, 193), which is highly expressed in patients with ALD (253). LMW-HA pro-inflammatory effects are mediated through activation of MMPs (321), plasminogen activator inhibitor (322), nitric oxide (323), and production of several pro-inflammatory cytokines including IL-8, IL-12, MIP-1α, MIP-1β, MCP-1 by macrophages (119, 120, 121, 122), and IL-1β, TNF-α, and IL-12 by DCs and endothelial cells (191). LMW-HA is considered an ECM-DAMP known to activate the NLRP3 inflammasome and subsequent cleavage of caspase- (126). The activation of the NLRP3 is induced by lysosomal digestion of LMW-HA into fragments upon internalization by macrophages *in vivo* and *in vitro*, a CD44-dependent process (196). Internalized LMW-HA interacts with the protein NLRP3 and induce release of IL-1β and CXCL2 chemokine.

In the liver, HA is synthesised mainly by HSCs (324), while degradation occurs principally by LSECs through HYAL1 and HYAL2 activity (324, 325). This produces LMW-HA that gathers at sites of active tissue catabolism and promotes inflammation. In a study by Mustonen and colleagues, the relationship between HA synthesis and degrading enzymes was demonstrated in histological stainings of liver sections from controls and patients with steatosis, steatohepatitis, cirrhosis and HCC (197), highlighting the importance of HA turnover in healthy liver and in CLD.

### Biglycan

Biglycan is a secreted member of the small leucine-rich proteoglycans (326, 327, 198). ECM-bound biglycan does not induce any immune response as it is incapable of activating TLRs. Proteolysis of ECM caused by stress or tissue damage releases soluble biglycan, the first endogenous proteoglycan described to interact with TLRs (198). Soluble biglycan generates a rapid inflammatory response that can lead to *de novo* synthesis of new biglycan. In macrophages, biglycan binds to TLR2 and TLR4 mediating innate immunity and leading to activation of p38, Erk, and NF-κB and finally to a pronounced expression of TNF-α and MIP-2. This evidence was confirmed in experiments with TLR4-mutant, TLR2^-/-^, and myeloid differentiation factor 88 (MyD88)^-/-^ macrophages, where the stimulatory effect of biglycan was significantly reduced, and in TLR2^-/-^/TLR4-mutant macrophages, that showed complete loss of activity (198).

Other downstream effector molecules involved in the signaling generated upon interaction of biglycan with TLR2/4 are IL-1β (199, 200), CXCL13 (200), MIP-1α, MCP-1, and RANTES (201). Biglycan is also considered an important link between the innate and adaptive immune response. In lupus nephritis, biglycan induces enhanced synthesis of CXCL13 which promotes recruitment of B1 cells, a subset of B cells involved in early and T-cell-independent antibody production (200).

Cleavage of biglycan by proteases can lead to generation of fragments; some of which are able to bind to TLR2/4. MMP-9 and MMP-12 are capable of degrading the ECM and generate specific biglycan-derived fragments that have been studied as biomarkers in liver fibrosis as they correlated with the severity of fibrosis, indicating that biglycan could mediate activation of sterile inflammatory responses (202).

### Elastin-Derived Peptides

Elastin is composed of soluble tropoelastin monomers which aggregate and cross-link to form the insoluble protein core of elastin fibres (328). Tropoelastin and elastin degradation products can influence inflammation and tissue remodeling (329). Despite crosslinking, physiological, pathological and aging-related ECM remodeling can lead to elastin degradation and production of soluble elastin-derived peptides (EDPs) or elastokines (330). MMP-2, MMP-7, MMP-9, MMP-12, and neutrophil proteases are enzymes actively involved in elastin degradation (329). Most studies focus on a single enzyme family-induced production of EDPs, hindering the identification of EDPs with shared or distinct biological properties. For instance, some generated EDP harbour a GxxPG consensus motif (where x represents any amino acid) essential for their bioactivity as it allows the interaction with the elastin-binding protein, a component of the elastin receptor complex (ERC) (203). EDPs generated from MMP-7, MMP-9, and MMP-12 contain the bioactive peptide VGVAPG, but differences in the number of peptides produced have been identified among the three MMPs (331). EDPs generated by neutrophil-derived serine proteases also included the bioactive GxxPG motifs VGVAPG, but other new bioactive motifs were also identified, such as GVYPG, GFGPG, and GVLPG (332). Here as well, different enzymes (human leukocyte elastase, proteinase 3, and cathepsin G) showed differences in respect to the number of peptides produced, their sequence and the cleavage site specificity and preferences. Some peptides produced by proteinase 3 and cathepsin G contained multiple bioactive motifs that may enhance biological activity of EDPs due to an increased probability of interaction with EDP (333). Whether these distinct peptides possess distinct biological properties has yet to be investigated.

EDPs are potent stimulators of tissue repair, but continuous exposure of cells to the elastokines can induce a chronic inflammatory state (334). EDPs are able to attract peripheral blood monocytes and polymorphonuclear leucocytes (335, 336), and induce changes in gene expression of inflammatory cells at the site of injury (3). They are able to stimulate T cells to release IFN- γ, which correlates with emphysema severity (124), and can drive polarization toward a Th-1 response (204). In monocytes, EDPs can modulate the expression of proinflammatory cytokines TNF-α, IL-1β, and IL-6 (123).

In the normal liver, elastin is a minor component of the ECM, but tropoelastin is actively synthesised by hepatic myofibroblasts in liver fibrosis regardless of the aetiology (337, 338). In liver cirrhosis, elastin fibre accumulation is found around the main branches of the hepatic artery and the portal vein (329) and correlates with adverse liver-related outcomes in patients with advanced CLD (339). Elastin deposition in patients with chronic viral hepatitis occurs concomitantly with the formation of thick collagen bands, providing a correlation between age of the scars and elastin content (340, 341, 342). Nevertheless, elastin turnover is detected in liver fibrosis and cirrhosis, indicated by the presence of elastin fragments in the serum and markers of degradation of mature cross-linked elastin in the urine (343, 344). EDP production during aging plays an important role in the development of NASH. In the inflammatory-aging process, enzymatic activity of neutrophil elastase increases, inducing a high release of elastokines (205). These EDPs bind to the ERC and participate to the development and progression of diseases such as cancer, atherosclerosis (345), insulin resistance (346), and lipid accumulation and inflammation in the liver (330). Neutrophil elastase is known to increase its activity in mouse models of obesity and in obese patients. This results in the production of EDPs that stimulate accumulation of hepatic triglycerides, cytokine expression, inflammation, and hepatic ECM remodeling leading to strong fibrosis and NAFLD to NASH transition (330).

### SPARC

Secreted protein, acidic and rich in cysteine (SPARC), or osteonectin, is a matricellular protein in the ECM that affects collagen fibre assembly and the activity of TGF-β1 (347) (4). SPARC is constitutively expressed in the normal liver (208) and is upregulated in activated HSCs (209), in rat and human cirrhotic liver (208, 210, 211) and in HCC (212–214). Its expression correlates with severity of liver fibrosis. Local production of SPARC was found to induce collagen deposition, inflammatory cell recruitment, TGF-β1 production, mesenchymal stem cell proliferation, and synthesis of ECM proteins in kidney, lung, and skin fibrogenesis (206, 207). The profibrogenic activity of SPARC has been connected to its ability to regulate TGF-β1 and collagen I synthesis (206). Decrease in TGF-β1 expression, obtained through siRNA silencing, decreased expression of SPARC; and decreased SPARC expression led to a decrease in TGF-β1 activity (348). In SPARC^-/-^ mice, the amount of CD4^+^ T cells was greatly decreased compared to wild type animals, and BDL in SPARC^-/-^ animals showed a strong decrease in collagen deposition when compared with control animals, possibly mediated by downregulation of TGF-β1 expression (349).

In a study by Mazzolini and colleagues, SPARC was detected in liver tissue samples of NAFLD patients, showing high levels of SPARC associated with higher mRNA levels of collagen Iα and TGF-β1 (350). They also showed that SPARC^+/+^ mice presented stronger inflammation than SPARC^-/-^ animals, with higher expression of pro-inflammatory cytokines and chemokines IL-6, CXCL10, and FAS/CD95 and increased infiltration of F4/80^+^ hepatic macrophages in response to high fat diet. Fibrosis, inflammatory cells and chemokines were significantly reduced in SPARC^-/-^ animals with hepatic injury induced by fatty acid accumulation, suggesting a role of SPARC in establishing a pro-inflammatory hepatic microenvironment.

Secreted modular calcium-binding protein 2 (SMOC2) belongs to the SPARC family of matricellular proteins and has also been suggested to be involved in fibrosis, inflammation, and cell differentiation and proliferation (351–353). SMOC2 has been identified as a key player in NAFLD progression. SMOC2 mRNA and protein levels were higher in liver samples of patients with NAFLD and in mice fed with high fat diet (354). SMOC2-knockdown mice fed with high fat diet showed lower collagen deposition compared to control animals, associated with a lower expression of TGF-β1 and α-SMA, and lower levels of pro-inflammatory cytokines IL-1β, IL-4, IL-6, MCP-1, and TNF-α in serum and liver samples. This study proved that SMOC2 ablation was protective against high fat diet-induced fibrosis and inflammation.

## ECM Proteins Which Drive Anti-Inflammatory Immune Responses

### Extracellular Matrix Protein-1

ECM-1 is a secreted glycoprotein that affects Th2 cell migration in asthma animal models and Th17 differentiation through interaction with CD122 receptor on T cells and αν integrins in experimental autoimmune encephalomyelitis (215, 216). It also regulates T follicular helper (TFH) cell differentiation (217). TFH cell differentiation in germinal centres is critical for their involvement in the production of high-affinity antibodies by activated B cells and plasma cells, and in mice it is positively regulated by IL-6 and IL-21 and suppressed by IL-2 (217, 355). IL-6 and IL-21 induce ECM-1 expression, which is a positive regulator of TFH cell differentiation in humoral immunity, germinal centre formation and antigen-specific antibody production by antagonising IL-2 signaling (217). Fan et al. described ECM-1 as a key mediator of liver tissue homeostasis (86). Depletion of ECM-1 severely affected liver architecture in mice, promoting liver fibrosis development, but without significant inflammation or hepatocyte damage (86). The authors found that ECM-1 was down-regulated in hepatocytes (and potentially other hepatic cell types) after liver damage, which activated TGF-β, probably *via* interacting with αν integrins, and releasing it from deposited latent TGF-β complexes to initiate HSC activation and fibrogenesis. Also, ECM-1 expression decreases in patients with liver fibrosis and inversely correlates with severity of fibrosis (86), but little is known about the regulation of the immune system by ECM-1 in CLD.

### High Molecular Weight-Hyaluronic Acid

High-molecular weight (HMW-HA) (>2,000 saccharides; >400 kDa) is abundant in uninjured tissues and in healing tissues, and is considered inert or anti-inflammatory (131, 356). HMW-HA has been described as an inhibitor of TLR2 signaling (192) and phagocytosis by monocytes (219), and limits antigen-antibody interactions (220).

HA is known to bind to CD44 and TLR2 expressed on immune and non-immune cells (218) (12). T-cells and monocytes interact with HA only if CD44 is activated by pro-inflammatory cytokines, such as TNF-α and IFN-γ, or by T cell receptor (TCR) triggering (357). HMW-HA promotes the expression of Foxp3 by activated Tregs through interaction with CD44 (118, 221) and is able to provide a positive environment to support the persistence and suppressive function of Tregs pre-activated *via* their TCR complex (118). This property, together with the ability of HMW-HA to induce death of activated T cells (358), supports the role of this ECM component in regulating the inflammatory milieu. Bollyky and colleagues also described that HMW-HA modulates inflammation promoting induction of CD4^+^Foxp3^-^ IL10-producing Treg (TR1) cells in peripheral tissues (218). These cells are important regulators of inflammation and maintain peripheral immunotolerance through the secretion of IL-10 (359, 360). HMW-HA was able to promote IL-10 production by murine CD4^+^Foxp3^-^ cells mainly *via* cross-linking of CD44. In fact, CD44^-/-^ mice showed a significantly lower up-regulation of IL-10 in response to HMW-HA. In human CD4^+^CD25^-^ T cells, co-stimulation with HMW-HA significantly increased IL-10 production and generated TR1 cells that demonstrated active prevention of inflammation in a colitis model.

Other CD44 ligands impact the HA-mediated effect on TR1 induction. OPN, a known CD44 ligand which impacts IL-10 production (240, 361) is found in abundance in chronic inflammation and is known to aggravate autoimmunity (362, 363). OPN inhibits HA-mediated TR1 induction and production and mRNA expression of IL-10, irrespective of IL-2 supplementation. These effects are mediated through interaction with both CD44 and integrin α_ν_β_3_ receptor, suggesting that OPN and HA are part of an intricate network of ECM components that regulate TR1 pathways.

### Proteoglycan 4

Proteoglycan 4 (PRG4), or lubrican, has been shown to promote an anti-inflammatory response in inflammatory osteoarthritis and rheumatoid arthritis (364). This proteoglycan interacts with TLR2, TLR4, and CD44 on synovial fibroblasts suppressing the production of a number of pro-inflammatory mediators and decreasing fibroblast proliferation (117, 222, 223, 224).

The liver presents high levels of PRG4 and the amount of PRG4 increases in response to high fat diet (225). PRG4 KO mice are protected from high fat diet-induced hepatic steatosis and exhibit an improved glucose tolerance and lower degree of white adipose tissue inflammation (225), suggesting a role for PRG4 in the regulation of nutritional homeostasis and inflammation in fatty liver disease.

## ECM Proteins With Both Pro- and Anti-Inflammatory Effects on Immune Cells

### Tenascins

The tenascin family includes four members: tenascin-C, tenascin-R, tenascin-X, and tenascin-W (365). Tenascin-C is a multifunctional and multimodular ECM glycoprotein that binds to a range of ECM proteins (95, 96) and binds receptors such as epidermal growth factor receptor (EGFR), TLR4 and integrins (96), as well as a wide range of growth factors [TGF-β, FGF, VEGF, PDGF, and insulin growth IGF-BPs (95, 96)]. The variety and number of interactions described for tenascin-C make it a multifunctional orchestrator of cell- and microenvironment-specific responses.

Tenascin-C is required for the inflammatory response to LPS *in vivo*. Piccinini et al. showed that, in a mouse model of experimental sepsis, tenascin-C^-/-^ mice showed delayed and less severe symptoms of sepsis after LPS injection compared to tenascin-C^+/+^ mice (230). The authors suggested that upregulation of tenascin-C is an early response to LPS-induced TLR4 signaling, and this glycoprotein appears to specifically control the switch from anti- to pro-inflammatory cytokine response downstream to TLR4 activation (230). Tenascin-C activation of TLR4 induces soluble pro-inflammatory mediators, such as IL-6, IL-8, and TNF, important mediators of T-cell polarization, in a number of cell types, including macrophages (82, 226, 227, 228), DCs (229), fibroblasts (226, 366) and chondrocytes (231). Similarly, in a murine model of arthritic joint disease, Ruhmann and colleagues demonstrated that tenascin-C deficient mice show presence of DCs with diminished pro-inflammatory cytokine release and reduced capacity to drive naïve T cells toward Th17, but not Th1, Th2, or Treg cell differentiation, in response to LPS stimulation (233).

Tenascin-C is also involved in modulation of inflammation upon sterile tissue injury *via* activation of TLR4 and integrins, including α_9_β_1_ and α_ν_β_3_, promoting tissue repair but also contributing to chronic inflammation (230). Upon tissue injury, tenascin-C contributes to both early inflammatory response and subsequent tissue remodeling by activating cell-type specific responses *via* TLR4 (96). Persistent expression of tenascin-C has been implicated in driving chronic inflammation in autoimmune, neurological, metabolic, and fibrotic diseases, where it is likely to activate a positive feedback loop further driving disease (96, 112). It is associated with erosive joint disease and poor response to biological treatment in patients with rheumatoid arthritis (367), whereas mice lacking tenascin-C are protected from prolonged synovial inflammation and tissue destruction in joint disease (226, 233). This sterile inflammation seems to originate from distinct modes of TLR4 activation and downstream signaling compared to pathogenic TLR4 agonists (227).

In the liver, tenascin-C knockout mice with liver fibrosis induced by concanavalin A showed protection against collagen deposition and inflammatory cell infiltration, reduced IFN-γ, TNF, IL-4, and TGF-β1 production, and a lower number of activated HSCs in respect to wild type animals (239). This study suggested that tenascin-C may support the recruitment and activation of lymphocytes with cytokine upregulation in immune-mediated hepatitis conditions. Tenascin-C upregulation was detected in a rat model of liver fibrosis induced by CCl_4_ (238), probably produced by HSCs (368) and in the livers of patients with liver cirrhosis, HCC and chronic HCV, where it correlates with progressive disease activity (82, 83, 84, 85). Plasma levels of tenascin-C, together with AST, also provide an effective model to discriminate HCV cirrhotic patients with active infection from healthy controls and patients with undetectable levels of HCV RNA post HCV treatment regimen, highlighting tenascin-C as a potential indicator of ongoing hepatic injury and inflammation in these patients (369).

Interestingly, tenascin-C can also have immunosuppressive effects through regulating local concentration and activity of the immunosuppressive factor TGF-β (112, 125) and through suppression of T-cell activation induced by various stimuli (232, 234–237). Whether this local regulation of TGF-β by tenascin-C directly affects hepatocyte or cholangiocyte senescence in acute or chronic liver injury is unknown. This evidence supports the dual nature of tenascin-C, where from one side it mediates tissue repair, suppressing excessive inflammation and regulating tissue remodeling, while in other conditions it drives chronic inflammation and promotes fibrogenesis. Key aspects behind this dichotomy could be temporal and spatial control of tenascin-C expression.

The largest member of the tenascin family, tenascin-X, is involved in collagen deposition, collagen fibrillogenesis and development and maintenance of elastic fibers (370, 371, 372). In a mouse model of NASH induced by high-fat and high-cholesterol diet, absence of tenascin-X reduced inflammation and liver dysfunction in response to the diet compared with control same diet-fed wild-type animals. This study suggested that tenascin-X may promote high-fat and high-cholesterol diet-induced liver dysfunction through enhancement of the inflammatory response. Since tenascin-X has been reported to interact with the latent form of TGF-β regulating its activation into active form (373), it was hypothesized that the lack of tenascin-X in knockout animals could attenuate the activation of TGF-β upon feeding high-fat and high-cholesterol diet. This reduced TGF-β activation may then result in suppression of the inflammatory response. Similarly to tenascin-C, tenascin-X also binds to integrins, including β1-containing and α_11_β_1_ integrins (373, 374) on the cell surface, but the correlation between tenascin-X-integrin signaling and hepatic dysfunction and inflammation has yet to be investigated.

### Osteopontin

OPN, also called secreted phosphoprotein-1 (SSP-1), is an extracellular structural glycoprotein that plays an important role in pathological conditions. Soluble and ECM-associated OPN are found in abundance in chronic inflammation, cancer and autoimmune diseases (218, 241). OPN binds to CD44 and integrin receptor α_v_β_3_ (218, 240), the main mediators of OPN activity. The canonical CD44 ligand is HA. In a study by Bollyky and colleagues, OPN inhibited IL-10 production through interaction with CD44 on TR1 cells (218). OPN was able to block HA induction of TR1 binding to CD44 and integrin receptors, highlighting the role of OPN in controlling TR1 pathways.

In the liver, elevated expression of α_v_β_3_ integrin is associated with high OPN levels in human alcoholic cirrhosis and mouse models of ALD (24). In addition, CD44 has been demonstrated to be involved in the regulation of inflammation in liver disease and is elevated in patients with ALD, including patients with alcoholic steatosis, alcoholic hepatitis and alcohol cirrhosis (253).

Initially, OPN was described in necrotic areas of the liver where it was contributing to local infiltration of KCs and resident macrophages (375). OPN has since been found to be involved in ALD, acute liver failure, NAFLD, liver fibrosis from HBV and HCV infection, and HCC (244–251), with increased circulating and ECM-associated OPN in patients with CLD. In animal models, OPN acts as a chemoattractant for hepatic resident macrophages and neutrophils (241, 242). OPN-deficient mice show impaired neutrophil infiltration, F4/80^+^ hepatic macrophage accumulation and release of pro-inflammatory cytokines (376).

Conversely, some studies suggest that OPN protects the liver from inflammatory injury, reducing production of IL-6, TNF-α, IL-1β, and toxic inducible nitric oxide synthase (iNOS) by F4/80^+^ hepatic macrophages, promoting hepatocyte survival (252). OPN deficiency in mice also protected against alcoholic hepatitis from chronic alcoholic steatohepatitis, showing increased induction of neutrophil infiltration in the alcoholic hepatitis model (377). In granuloma formation upon liver injury, OPN initially induces IL-12 and IFN-γ synthesis, while at late stages, OPN increases recruitment of CD4 T cells, F4/80^+^ macrophages and DCs into the liver and enhances TNFα production (243). In liver fibrosis, OPN correlates with disease severity and progression of disease (241). OPN can activate HSCs *via* CD44 (253) and *via* α_v_β_3_ integrin resulting in upregulation of collagen I and transdifferentiation of HSCs (378). In patients with chronic liver fibrosis, hepatic OPN expression correlates with high TGF-β1 expression, portal space neutrophil-related inflammation and portal hypertension (248, 254). Portal inflammation is also predicted by high serum levels of OPN in patients with NAFLD (246). Mice treated with an OPN-neutralizing antibody show reduced obesity-driven hepatic inflammation and F4/80^+^ and Mac-2^+^ hepatic macrophage accumulation (379). In NASH, OPN produced by NKT cells induces activation of HSCs and progression of fibrogenesis (380).

The mechanism behind the dual role of OPN has not been specifically investigated, but it could be pathophysiological-dependent, spatiotemporal-dependent or cell/receptor-dependent. For example, OPN expression during early or late stages of inflammatory diseases seems to regulate the production of cytokines with distinct pro- or anti-inflammatory functions (381, 382, 383). Different isoforms, terminal fragments and post-transcriptional modifications of OPN may yield different impact as described in acute brain injury (384, 385, 386). Finally, OPN binds to multiple integrins and other receptors on multiple cell types, possibly mediating distinct phenotypic changes (387).

### Collagen

Collagens are a large family of ECM proteins with 29 types, all characterized by a right-handed triple helix structure composed of three polypeptide chains. Receptors that bind directly to collagen have been identified on the surface of several cell types, e.g., α_1_β_1_, α_2_β_1_, α_10_β_1_, and α_11_β_1_ integrins. Hepatocytes and other cell types express the integrin receptor, α_1_β_1_ (255), which can bind to collagens I, III, IV, IX, XIII, XVI, and IV chain-derived peptide arresten (388, 389, 390). Other collagen-binding receptors include osteoclast-associated immunoglobulin-like receptor (OSCAR), known to have a role in the maturation of monocyte-derived DCs (258), and G-protein-coupled receptor (GPR) 56, present on fibroblasts, oligodendrocytes, melanoma cells, and cytotoxic NK and T lymphocytes (259, 260, 261). In addition, collagens I, II, III, and XVII are high-affinity ligands of leukocyte-associated immunoglobulin receptor-1 (LAIR-1 or CD305), expressed in both mice and humans on numerous immune cells, including NK cells, T cells, B cells, monocytes/macrophages, DCs, eosinophils, basophils, and mast cells (263). LAIR-1 is an inhibitory immune cell surface receptor which plays a key role in regulating inflammatory responses and LAIR-1:collagen interaction confers a general immunosuppressive activity (115, 256, 391). While intrahepatic immune cells are not exposed to collagens physiologically, collagens are ECM ligands that directly down-regulate immune responses through LAIR-1.

Surface-conjugated LAIR-1 significantly inhibits production of pro-inflammatory signaling molecules such as monokine induced by IFN-γ, MIP-1α, MIP-1β, MIP-2, and RANTES (256). Cross-linking of LAIR-1 with monoclonal antibodies or its ligands, inhibits the cytotoxic activity of NK and CD8^+^ T cells (392), differentiation and activation of B cells (393), the T cell receptor/CD3 complex signaling (394), the differentiation of peripheral blood precursors into DCs (395, 396) and the production of type-I interferon by CpG-activated plasmacytoid DCs and monocytes (397). Chronic autoimmune inflammation has also been linked to low expression of LAIR-1 (398).

Regulation of LAIR-1 is obtained by modulating its expression on the surface of immune cells during differentiation and activation (393, 394, 262, 399, 400). LAIR-1 can also be shed from immune cells upon cell activation (401) and can be regulated through competition with LAIR-2. LAIR-2 is a secreted protein that interacts with the same collagen sequences as LAIR-1, functioning as the decoy counterpart of LAIR-1. This soluble competitor to LAIR-1/collagen immunosuppressive activity (262) has been detected in primary PBMCs, monocytic, and T cell lines. PBMCs show increased LAIR-2 expression upon stimulation with phorbol 12-myristate 13-acetate (PMA) and ionomycin, mainly produced by CD4^+^ T cells (262). The affinity for collagens I and III is comparable between the two receptors, suggesting that LAIR-2 may function as a pro-inflammatory mediator *in vivo* by decreasing the inhibitory potential of the immune inhibitor LAIR-1. In contrast, a reduced concentration of LAIR-2 may result in LAIR-1 binding to collagens, repressing immune cell activity at sites where it is not needed. Recently, the role of LAIR-1 expression on monocytes and macrophages in the development and progression of liver cirrhosis was studied in liver biopsies and peripheral blood samples of cirrhotic patients (116). LAIR-1 was expressed in hepatic resident CD68^+^ macrophages, and the number of LAIR-1-positive cells was reduced in cirrhotic livers. On the contrary, LAIR-1 was highly expressed in peripheral blood monocytes and their expression level of LAIR-1 [median fluorescence intensity (MFI)] was higher in cirrhotic patients than in healthy controls, in particular in the intermediate subset of monocytes (CD14^++^CD16^+^). These data suggest a role for LAIR-1/and LAIR-2/collagen interactions in the regulation of different immune cells involved in inflammatory responses in CLD.

### Decorin

Decorin is a member of the small leucine-rich proteoglycans family. The protein core can interact with several receptor tyrosine kinases including EGFR (402), VEGF receptor (VEGFR) 2 (403), and with TLR2 and TLR4 receptors, evoking pro- and anti-inflammatory effects and recruiting macrophages (264, 265). Decorin is also a ligand for α_2_β_1_ integrin (266), widely expressed on epithelial and endothelial cells, fibroblasts, T-cells, myeloid cells, and others, with a role in inflammation during rheumatoid arthritis.

KO decorin mice show an increase in inflammation and fibrogenesis (79, 404, 405), while administration of recombinant decorin and decorin gene therapy showed anti-inflammatory effects (79, 406). Nevertheless, interaction between decorin and TLR2 and TLR4 on peritoneal macrophages triggers an acute inflammatory reaction with activation of p38, mitogen-activated protein kinase (MAPK), and NF-κB, increased synthesis of pro-inflammatory cytokines TNF-α and IL-12, and inhibition of anti-inflammatory IL-10 (265). This dual function is the result of ECM remodeling: cleavage of decorin by MMPs and other proteases can generate fragments of decorin which can act as ECM-DAMPs through TLR2 and TLR4, turning anti-fibrotic decorin into peptides with pro-inflammatory and pro-fibrotic activity (79, 117, 269).

In the liver, decorin accumulates along the sinusoidal walls (407), co-localizes with large amounts of TGF-β1 in fibrotic areas in chronic hepatitis and cirrhosis (408), and is upregulated during NASH progression (270).

### Fibronectin

FN is the most abundant ECM glycoprotein in the liver and exists as several different forms. pFN, its soluble form, is synthesised by hepatocytes and assembled into the ECM where it can bind a plethora of growth factors. cFN, is found at low levels in the liver (24), but accumulates in response to injury and pathological stimuli (271). cFN contains an additional type III extra domain A and B (EDA^+^FN and EDB^+^FN). THP-1 monocyte/macrophages show increased MMP-9 production in response to recombinant EDA and EDB (409). EDA, in particular, was found to activate macrophages *via* TLR4, in a MD-2 (a TLR4 accessory protein) co-expression dependent manner. EDA-containing cFN activation of macrophages *via* TLR4 was confirmed in two studies by Doddapattar and colleagues, which showed that EDA^+^FN increased expression and activity of MMP-9 and potentiated dose-dependent NFκB-mediated inflammation (including secretion of TNF-α and IL-1β) in bone marrow–derived macrophages from atherosclerotic apolipoprotein E–deficient (Apoe^−/−^) mice (272). EDA-containing FN fragments are widely recognized as ECM-DAMPs, and are upregulated in several inflammatory diseases (myocardial infarction, cardiac fibrosis, stroke, cerebral ischemia, airway fibrosis, dermal fibrosis, liver fibrosis), where they activate both immune and non-immune cells prolonging inflammation and fibrosis (271, 274, 409–417). In dermal fibrosis, dermal fibroblasts recognize EDA *via* α_4_β_1_ integrin and TLR4, initiating a fibro-inflammatory response that includes synthesis of cytokines IL-10 and IL-13, and enrichment in the ratio of EDA^+^FN: total FN, indicating that EDA^+^FN further stimulates production of EDA^+^FN (271).

In the liver, FN was identified as an early matrix molecule that links TLR4 activation of HSCs with LSEC angiogenesis in a BDL mouse model of fibrosis (274). cFN was also identified as a biomarker of early stage ALD (275), appearing in the liver during the first 8 weeks of ethanol feeding in a rat model. Furthermore, in a rat model of ALD, Aziz-Seible et al (273*)*. showed that KCs stimulated by exogenous cFN produced the pro-inflammatory cytokines, TNF-α and IL-6, as well as pro-fibrogenic factors, MMP-2 and TIMP-2.

Although FN is deposited during fibrosis and TLR4 is expressed on numerous liver cells including LSECs, KCs and HSCs, information regarding the potential role of EDA-containing FN fragments in regulating inflammatory microenvironments *via* TLR4 in CLD is limited.

## Other ECM Proteins Involved in CLD

### Laminin

Despite the lack of evidence for a direct effect on the immune system, laminin fragment P1 (LAM-P1) serum level, generated by pepsin digestion (277), was found to correlate with the degree of fibrosis from perivenular fibrosis to septal fibrosis to cirrhosis, and with inflammation, in patients with ALD (278). This evidence suggests that laminin and laminin fragments produced upon enzymatic digestion could be involved in regulation of the inflammatory progression in CLD. In the liver, only the β2 laminin chain is expressed in the sinusoids, portal biliary ducts, portal vascular structures and central veins, while other laminins are rare or absent (418). In liver fibrosis, deposition of laminin in the basement membrane increases together with that of type IV collagen and perlecan (418). Serum laminin level also correlates with degrees of fibrosis in alcoholic patients (419), it increases in cirrhosis in comparison to healthy controls (420), and in cirrhotic NAFLD patients compared to patients without cirrhosis (421).

## Discussion

In this review, we summarize evidence that specific intact ECM components and enzymatically digested ECM fragments act as ECM-DAMPs and are able to modulate the immune system during inflammation *in vitro* and *in vivo*. Increasing understanding of the signaling pathways involved in sensing and responding to these ECM molecules highlights the key role of the ECM and its remodeling processes in the fibrogenic evolution of CLD. The paucity of curative therapies for inflammatory/autoimmune liver diseases necessitates further understanding of the mechanisms that exacerbate the disease condition, and focusing on the ECM-immune system crosstalk in CLDs may help identify new pathways and ultimately better targets for therapeutic interventions. Direct targeting of the ECM involved in the activation and actuation of pro- and anti-inflammatory/fibrogenic activities mediated by the ECM could represent fibrosis-specific therapy which remains a clinically unmet need. Although most of the proteins included in this review have been identified in liver fibrosis and correlate with different aetiologies and disease stage, direct protein-immune cell interactions remain poorly explored. Their immunomodulatory activity in other inflammatory diseases suggest that they could be the main protagonists in orchestrating localized tissue-specific inflammatory environments typical of CLDs.

ECM proteins have been studied in human samples and in different *in vivo* models of fibrosis and cirrhosis, particularly in NAFLD/NASH. Among them, matricellular proteins CCN1 and periostin appear to have a strong link with initiation of NAFLD and progression to NASH. CCN-1 is an important regulator of inflammation in NAFLD mouse models, but its overexpression also correlates with increased myofibroblast senescence, indicating that further studies should be directed to investigating CCN1 involvement in progression of NAFLD, particularly in the transition to NASH. Periostin and ECM-DAMPS EDPs are also involved in the progression of NAFLD to NASH in mouse models and have been linked to the inflammatory stage of NAFLD in patients. Since these proteins showed strong effects on T cells in other inflammatory diseases, and on macrophage infiltration in hepatic fibrosis, a deeper understanding of periostin and EDP involvement in regulating these immune cells in NAFLD livers could provide new insights on disease progression.

Other proteins such as TSP-1, OPN, proteoglycans lumican and agrin, and matricellular proteins SPARC and SMOC2 have a clear association with fibrosis in NAFLD patients and animal models. Their ability to drive inflammation and fibrosis is mainly indirect, modulating release and activation of TGF-β1. Other mechanisms of action such as recruitment of macrophages and regulation of T cell activity seem to be involved in the regulation of inflammatory processes by these proteins, but the direct protein-immune cell interaction remains unexplored in the context of CLDs and needs further investigation in specific liver disease models.

ECM-decorin also modulates several growth factors and inhibits TGF-β1 activity, but its role in orchestrating inflammation is controversial. Decorin has been described as capable of stimulating both pro- and anti-inflammatory responses. This duality could derive from differences in signaling pathways induced by the intact protein or upon ECM remodeling that produces decorin fragments, ECM-DAMPs with known pro-fibrotic and pro-inflammatory properties. Given the conflicting evidence regarding decorin activity, more studies need to focus on understanding its dynamic role in CLD. Similarly, evidence supports the dual nature of tenascin-C, where from one side it mediates tissue repair, suppressing excessive inflammation, and regulating tissue remodeling, while in other conditions it drives chronic inflammation and promotes fibrogenesis. In this case, key aspects behind this dichotomy could be temporal and spatial control of tenascin-C expression and investigation of tenascin-C presence and kinetics in different phases of CLD could provide important information regarding its role in regulating the microenvironment upon liver injury.

Only PRG4 and ECM-1 demonstrated protection from steatosis and inflammation in high fat diet mouse models, and an inversed correlation with severity of liver fibrosis, respectively. These proteins are able to both interact directly with immune cells through surface receptors (evidence from other inflammatory diseases) and indirectly by regulating local cytokine microenvironments and activation of TGF-β1. Given their protective role against liver disease, further studies are required to elucidate how these proteins interact with other components of the ECM, their dynamic turnover and whether they reveal immunomodulatory properties in CLDs.

Local TGF-β concentration at tissue level is a key component of the microenvironment and its tight regulation is a main participant in liver homeostasis, regeneration and response to injury. Although different ECM proteins are able to modulate or activate local TGF-β, it is not known whether a correlation between ECM remodeling, TGF-β activation, and induction/amplification of hepatic cell senescence could play a role in CLDs and this link should be further explored.

The study of specific ECM changes and relative effects on immune cells is significantly less frequent in autoimmune hepatitis or other autoimmune diseases and ALD, where inflammation has a central role in disease progression. Increased expression of tenascin-C, OPN and TSP-1 is associated with autoimmune diseases, while collagen immunosuppressive receptor LAIR-1 decreases in chronic autoimmune disease.

Very little is known regarding deposition and remodeling of specific ECM proteins in ALD; while cFN is a biomarker for early disease stages, high OPN levels in the liver, associated with elevated expression of receptors α_v_β_3_ integrin and CD44, are detected in human alcoholic cirrhosis and mouse models of ALD. Some ECM-DAMPs with clear pro-inflammatory activities (biglycan fragments, EDPs, LAM-P1) were found to correlate with severity of fibrosis and inflammation in ALD and NAFLD/NASH, while others (LMW-HA, EDA-FN fragments, biglycan fragments, HS fragments) have not been identified or studied in CLDs. Since these ECM-DAMPs are able to bind to TLRs and derive from ECM proteins that are normally present in the liver and that change in proportion during hepatic fibrosis and ECM remodeling, their release and turnover should be specifically explored in CLDs to investigate whether they are actively involved in mediating inflammatory microenvironments.

ECM-DAMP involvement has been shown in a large number of liver inflammatory conditions. During the last 20 years, several mechanisms of ECM-DAMP generation, receptors and signaling pathways have been identified and they represent promising targets in the treatment of inflammatory conditions. However, there are still several gaps in knowledge that make translation of this field to effective therapies unsatisfactory. Our current knowledge regarding ECM-derived DAMPs, is still quite primitive, and it seems clear that there are many more types of DAMPs awaiting description. In our opinion, several key questions need to be addressed that concerns ECM-DAMPs, including: 1) what triggers ECM-DAMP release from the ECM and/or *de novo* synthesis upon tissue injury? 2) How do different ECM-DAMPs interact. 3) What ECM-DAMPs have additive effects or compete for binding to the same PRR (e.g., HA and OPN)? 4) The importance of timing and ECM-DAMPs local concentration on the interaction with PRRs and the downstream effects. 5) Do ECM-DAMPs and PAMPs interact during PAMP-dependent inflammation? The liver is exposed to microbial products from the gut and this translocation can impact the progression of CLD. ECM remodeling and release of ECM-DAMPs upon liver injury may directly stimulate sterile inflammation that in turn may have an effect on PAMP-induced activation of immune cells.

A potential avenue that should be further pursued is the use of ECM-DAMPs as biomarkers that might have diagnostic relevance in different aetiology and stages of CLD. ECM-DAMPs could represent non-invasive measures to facilitate the diagnosis of liver injuries and assess the fibrogenic evolution of CLD. Assessment of liver fibrosis is important to monitor the progression of CLD to end-stage cirrhosis, plan surveillance strategies and implement therapies. Quantifying serum biomarkers originated from ECM remodeling could be a valuable tool, in combination with other non-invasive methods, to overcome the disadvantages inherent in the use of liver biopsy approach, the recommended standard method for histological assessment of liver fibrosis. The potential use of ECM-DAMPs as biomarkers of liver disease stage and outcome prediction is supported by studies that highlight the detection of some products of ECM remodeling in the serum of patients. Examples were represented by elastin fragments in the serum of fibrotic and cirrhotic patients, the correlation of LAM-P1 serum level with the degree of fibrosis progression and inflammation in ALD and NAFLD patients, and the high levels of biglycan in the serum of rat models of liver fibrosis. Despite these advances, there is much that still could be addressed. Other ECM-DAMPs are likely to hold similar diagnostic or prognostic value if analysed in the appropriate animal model or cohort of CLD patients. For instance, EDA-containing and decorin fragments have demonstrated direct modulation of fibrosis; studies of their correlation with fibrosis stage or aetiology could reveal useful information for clinical application.

Moving forward, given the important role of the ECM in CLD, this central feature of the microenvironment needs to be taken into account when pathways of disease development and progression are studied. Conventional 2D *in vitro* models made of cell cultures on glass or plastic, are usually too simplified in terms of number and complexity of cell populations and microenvironment. This approach ignores the important interactions of hepatic and immune cells with the surrounding microenvironment (including the ECM) *via* integrins and other ECM-receptors, excluding related signaling pathways from the equation. On the other side, studies in animal models certainly present the advantage of using a physiologically relevant model that includes the ECM and cell-matrix interactions, but they are also associated with ethical concerns, limited reliability for predicting human responses to treatments, substantial differences in anatomy, immune responses, metabolic activity, and development in respect to humans, and low reproducibility (422). Most importantly, mechanistic studies on ECM components usually utilize specific -/- animals for a target protein, obtaining an “all or nothing” effect that does not translate into the complexity of human disease in terms of timing and overall effect when in presence of other compensatory mechanisms.

3D *in vitro* models may have the right features to provide more reliable platforms to understand health and disease biology in a more cost-effective, scalable and reproducible way than *in vivo* models. 3D cultures have demonstrated to be more effective in reproducing *in vivo* responses and cellular behaviours, as thoroughly reviewed by McCrary and colleagues (422). Given the critical role of the ECM in healthy tissue function and disease development and progression, incorporation of the ECM in these 3D culture models is essential to create the appropriate tissue microenvironment.

The lack of reliable and relevant 3D models of CLD that include the ECM and the immune system has hampered the study of the properties of the ECM. In addition, the tempo-spatial 3D signaling originated from the ECM and its remodeling should be considered when culture models are used to study inflammatory diseases such as CLD, e.g., generating and/or validating 3D models containing hepatic ECM from different stages of fibrosis. These models should include specific scaffolds with relative stiffness, combined with hepatic cells actively involved in the fibrotic process, and immune cells. To mimic the complex biochemical, physical and architectural features of the tissue microenvironment, one particularly promising approach is the use of native ECM as a biomaterial to develop 3D models, through a process called decellularization. Decellularization describes the treatment of a tissue or whole organ with a series of enzymes and detergents to effectively remove the native cellular content and DNA, while maintaining the ECM network. Decellularized scaffolds are tissue- and function-specific based on their origin and can be combined with different cell populations to create 3D models (423–425).

Decellularized ECM-scaffolds have demonstrated promising results in maintaining the native architecture and protein network for investigation of cell-to-matrix interaction. The advantages of using decellularized ECM-scaffolds in studying ECM composition and architecture and in disease modeling are vary; among the most important: i) decellularization eliminates the risk of negative immune responses due to removal of cellular content, making the scaffold “immuno-compatible” in the case of transplantation or co-culture with immune cells; ii) the ECM-scaffolds provide a natural template for the study of ECM composition using protemics profiling; iii) cell binding-sites are preserved in the ECM-scaffold, allowing for cell adhesion and studying of cell-ECM interactions; iv) disease and patient-specific phenotypes can be created using patient-derived tissue samples, replicating extremely specific microenvironments that may be difficult to be recreated by synthetic or more simple biomaterials.

**Figure 3:**
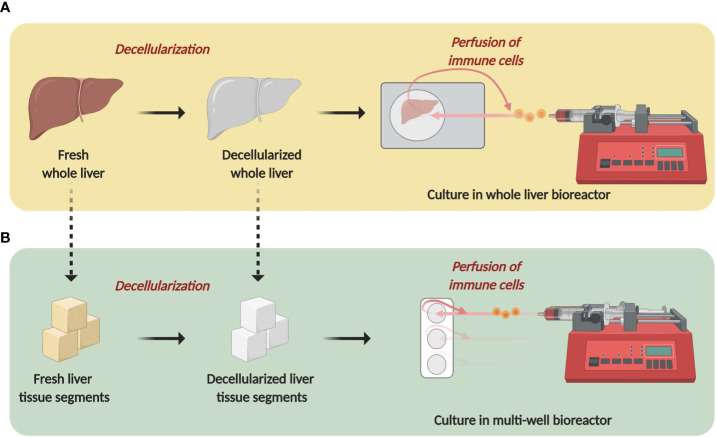
3D-dynamic perfusion-based immune competent systems to study extracellular matrix (ECM). Development of 3D-dynamic immune competent models to study ECM properties *in vitro*. **(A)** whole liver decellularization generates an acellular scaffold with maintained vasculature and ECM network. The resulting liver scaffold can be placed in a bioreactor which allows continuous circular perfusion of immune cells through the decellularized liver vasculature, facilitating immune cell:ECM interaction and monitoring of cellular phenotypes. **(B)** segments of liver tissue can be obtained from patient liver tissue and decellularized to generate acellular liver segments. These can be cultured in a multi-well bioreactor which supports perfusion of immune cells. The multi-well set up allows for direct comparison between eg healthy vs diseased tissue perfused with the same immune cells. Schematic was created using BioRender (https://app.biorender.com).

The presence of a 3D ECM, as in the case of 3D models currently in use such as decellularized scaffold-based bioengineered constructs (423, 426, 427) and precision cut liver slices (428, 429), provides microenvironmental cues that could potentially participate in/modulate the signaling pathways relative to the scientific question studied. Most importantly, given the importance of the immune system in CLD and its interaction with the remodeling ECM, organ- and disease-specific immune cells should be integrated into 3D models for the study of ECM remodeling in CLD. Furthermore, bioreactors have been developed to support 3D cultures providing fluid-flow and active mechanical stimulation to recapitulate the physiological environment which cells are exposed to and that plays an important role in fibrosis pathogenesis. Integration of bioreactors and micro/macrofluidic devices can also improve longevity and function of 3D models and provide temporal control over cellular microenvironment (430) (**Figure 3**).

Although combinations of 3D microenvironment, immune cells, hepatic cells and a bioreactor-based culture are still rarely used in research around the study of ECM remodeling and CLD mechanisms, such complex multicellular dynamic 3D models are starting to emerge (431, 432). One example is a recent work published by Collin de L’Hortet et al. that described the transition from NAFLD to NASH in an *in vitro* model developed seeding decellularized rat livers with human mesenchymal cells, endothelial cells, fibroblasts, macrophages and induced pluripotent stem cell (iPSC)-derived hepatocytes (431). In this model, liver inflammation, lipid peroxidation and lipid accumulation in NAFLD were studied upon manipulation of deacetylase sirtuin-1 (SIRT1) to identify its involvement in NAFLD progression to NASH. The 3D model was maintained in culture by a perfusion bioreactor and included key disease features, showing the full potential of tissue engineered ECM-based models to study mechanisms of disease progression.

Despite the clear complexity in generating such models, development of physio-pathologically relevant multi-layered disease-specific culture systems is imperative to understand the role of the ECM in establishing inflammatory microenvironments.

The use of decellularized ECM-scaffolds in *in vitro* models is not exempt from limitations: limited mechanical instability and significant altered mechanical properties in comparison to the native counterpart; batch to batch variability due to variations in the decellularization process among studies, donor gender, sex, genetics, treatment, and anatomical tissue sampling; difficulties in assessing the exact protein content in different ECMs (422). Finally, most decellularization techniques, especially if not properly validated, inevitably removes some growth factors and ECM proteins from the matrix. To address this specific problem, highly informative advanced proteomic techniques have been recently employed to understand tissue-specific ECM protein and growth factor composition, which can be used to develop relevant *in vitro* models of disease and to gain insights to the ECM remodeling in disease. ECM composition analysis through proteomic profiling of decellularized tissues potentially removes interference from intracellular content. The type and number of ECM proteins that can be identified in a proteomic screen depend on several variables, including the reproducibility and reliability of the decellularization method, and parameters of the proteomic process such as the protocol to digest proteins into peptides, the extent of protein or peptide fractionation, and the mass spectrometry acquisition parameters (10). Furthermore, due to the destructive nature of the solubilization methods and the sampling of organs and tissues, proteomics does not provide information regarding specific regions and distribution of ECM proteins. Different preparation protocols for the creation of decellularized ECM-scaffolds diversely effect matrisome compositions, as described by a recent paper published by Daneshgar and colleagues (30). In this study, it was observed that the human liver matrisome constitutes of many relatively lower abundant matrisome-associated proteins, and that different preparation protocols influenced more the matrisome-associated fraction than the overall core matrisome.

In conclusion, ECM molecules and ECM-DAMPs and the signaling cascades that they activate represent new targets in potentially halting, reducing or reversing fibrogenesis and concomitant inflammatory state in CLD. ECM remodeling and its effects on the immune system should be studied in advanced dynamic 3D models that contain hepatic ECM. ECM proteins and fragments up- or down-regulated during CLD development and progression should be further investigated for their key role in modulating the immune system.

## Author Contributions

CM and LU were involved in conceptualization, writing of the original draft, reviewing and editing. SC and RW were involved in reviewing and editing. All authors contributed to the article and approved the submitted version.

## Conflict of Interest

The authors declare that the research was conducted in the absence of any commercial or financial relationships that could be construed as a potential conflict of interest.

## Glossary

**Table:**

ALD	alcoholic liver disease
ALT	alanine transaminase
AST	aspartate aminotransferase
BDL	Bile duct ligation
BMDM	bone marrow derived macrophage
BMP-1	bone-morphogenetic protein-1
CCL	chemokine ligand
CCN1	cysteine-rich protein 61 or Cyr61
cFN	cellular fibronectin
CLD	chronic liver disease
CTGF	connective tissue growth factor
CXCL	chemokine (C-X-C) ligand
DAMPs	damage-associated molecular patterns
DCs	dendritic cells
DDA	DNA damage response
DDC	3,5 diethoxycarbonyl – 1,4- dihydrocollidine
ECM-1	extracellular matrix protein-1
ECM	extracellular matrix
EDA and EDB	extra domain A and B
EDPs	elastin-derived peptides
EGFR	epidermal growth factor receptor
ERC	elastin receptor complex
FGF-2	fibroblast growth factor-2
FN	fibronectin
GAG	glycosaminoglycan
GPR56	G-protein-coupled receptor 56
HA	hyaluronic acid
HBV	hepatitis B virus
HCC	hepatocellular carcinoma
HCV	hepatitis C virus
HGF	hepatocyte growth factor
HMW-HA	high molecular weight hyaluronic acid
HS	heparan sulphate
HSCs	hepatic stellate cells
HYALs	hyaluronidases
IFN	interferon
IGF-BPs	insulin growth factor-binding proteins
IL	interleukin
Inos	inducible nitric oxide synthase
IP	inflammatory protein
Ipsc	induced pluripotent stem cell
KCs	Kupffer cells
KO	knockout
LAIR-1 and LAIR-2	leukocyte-associated immunoglobulin receptor-1 and 2
LAM – P1	laminin fragment P1
LAP	latency associated protein
LLC	large latent complex
LMW-HA	low molecular weight hyaluronic acid
LPS	lipopolysaccharide
LRP	low density lipoprotein receptor-related protein
LSECs	liver sinusoidal endothelial cells
LTBP	latent TGF-β1-binding protein
MAPK	mitogen-associated protein kinase
MCP-1	monocyte chemoattractant protein-1
MDSC	Myeloid-derived suppressor cell
MDSCs	myeloid-derived suppressor cells
MFI	median fluorescence intensity
MIP	macrophage inflammatory protein
MMPs	matrix metalloproteinases
MyD88	myeloid differentiation factor 88
NAFLD	non-alcoholic fatty liver disease
NASH	non-alcoholic steatohepatitis
NK	natural killer
OPN	osteopontin
OSCAR	osteoclast-associated immunoglobulin-like receptor
PAMP	pathogen associated molecular pattern
PBC	primary biliary cholangitis
PBMCs	peripheral blood mononuclear cells
PDGF	platelet-derived growth factor
pFN	plasma fibronectin
PMA	phorbol 12- myristate 13- acetate
PRG4	proteoglycan 4
PRRs	pattern recognition receptors
PSC	primary sclerosing cholangitis
RANTES	regulated upon activation normal T cell expressed and secreted
RGD	Arg-Gly-Asp
ROS	reactive oxygen species
SASP	senescence-associated secretory phenotype
SIRT1	sirtuin 1
SMOC2	secreted modular calcium-binding protein 2
SPARC	secreted protein, acidic and rich in cysteine
SSP-1	secreted phosphoprotein-1
TCR	T cell receptor
TFH	T follicular helper
TGF-β	transforming growth factor-β
Th	T helper cell type
TIMPs	tissue-inhibitors of matrix metalloproteinases
TLR	toll-like receptor
TNF-α	tumor necrosis factor-α
TR1	Foxp3^–—^IL-10-producing Treg cells
Treg	regulatory T cell
TSLP	thymic stromal lymphoprotein
TSP-1	thrombospondin-1
VEGF	vascular endothelial growth factor
VEGFR2	vascular endothelial growth factor receptor 2
WISP1	Wnt-1-induced secreted protein 1
YAP/TAZ	yes-associated protein/transcriptional coactivator with PD2-binding motif
α-SMA	alpha-smooth muscle actin.
